# Detection of Bacteremia in Surgical In-Patients Using Recurrent Neural Network Based on Time Series Records: Development and Validation Study

**DOI:** 10.2196/19512

**Published:** 2020-08-04

**Authors:** Hyung Jun Park, Dae Yon Jung, Wonjun Ji, Chang-Min Choi

**Affiliations:** 1 Department of Pulmonary and Critical Care Medicine Asan Medical Center University of Ulsan College of Medicine Seoul Republic of Korea; 2 Big Data & AI Lab Hana Institute of Technology Hana TI Seoul Republic of Korea

**Keywords:** deep learning, bacteremia, early detection, time series, recurrent neural network, neural network, informatics, surgery, sepsis, modeling

## Abstract

**Background:**

Detecting bacteremia among surgical in-patients is more obscure than other patients due to the inflammatory condition caused by the surgery. The previous criteria such as systemic inflammatory response syndrome or Sepsis-3 are not available for use in general wards, and thus, many clinicians usually rely on practical senses to diagnose postoperative infection.

**Objective:**

This study aims to evaluate the performance of continuous monitoring with a deep learning model for early detection of bacteremia for surgical in-patients in the general ward and the intensive care unit (ICU).

**Methods:**

In this retrospective cohort study, we included 36,023 consecutive patients who underwent general surgery between October and December 2017 at a tertiary referral hospital in South Korea. The primary outcome was the area under the receiver operating characteristic curve (AUROC) and the area under the precision-recall curve (AUPRC) for detecting bacteremia by the deep learning model, and the secondary outcome was the feature explainability of the model by occlusion analysis.

**Results:**

Out of the 36,023 patients in the data set, 720 cases of bacteremia were included. Our deep learning–based model showed an AUROC of 0.97 (95% CI 0.974-0.981) and an AUPRC of 0.17 (95% CI 0.147-0.203) for detecting bacteremia in surgical in-patients. For predicting bacteremia within the previous 24-hour period, the AUROC and AUPRC values were 0.93 and 0.15, respectively. Occlusion analysis showed that vital signs and laboratory measurements (eg, kidney function test and white blood cell group) were the most important variables for detecting bacteremia.

**Conclusions:**

A deep learning model based on time series electronic health records data had a high detective ability for bacteremia for surgical in-patients in the general ward and the ICU. The model may be able to assist clinicians in evaluating infection among in-patients, ordering blood cultures, and prescribing antibiotics with real-time monitoring.

## Introduction

Bacteremia is associated with increased morbidity and mortality [1]. As bacteria grow in the bloodstream, subsequent immune response can cause sepsis, a life-threatening organ dysfunction [2]. Early administration of antibiotics is important for reducing the mortality associated with this infectious condition [3]. As the observable features of infection are symptoms or laboratory data, the systemic inflammatory response syndrome (SIRS) criteria have been used to detect sepsis in bedside medicine [4], even though it harbors issues such as underscoring and inaccuracy [2]. Variations of rule-based scoring systems such as the Modified Early Warning Score (MEWS) [5] and the Sequential Organ Failure Assessment (SOFA) [6] have also been developed; however, these systems are not commonly used outside the setting of critical care [2].

More recently, machine learning or deep learning–based models that use data from electronic health records (EHRs) were developed for early prediction of sepsis [7-9]. However, labeling the start time of sepsis is a delicate matter. Different scholars have used different criteria to define sepsis, such as the increase of SOFA score [8] or the detection of any two SIRS criteria in in-patients with the International Classification of Diseases, Ninth Revision (ICD-9) codes of sepsis [7,9,10]. The SIRS criteria should only be used when an infection is suspected [11]. This is why the ICD-9 code for sepsis is used with SIRS criteria; however, using the ICD-9 code does not guarantee that the patients are indeed suspected with sepsis, especially if other interventions such as surgery are performed. Another gold standard of defining sepsis is the Sepsis-3 criteria, which refers to the increase of the SOFA score, that is also used in suspected infection. Considering the infectious condition, some studies used a time stamp of antibiotics and blood culture as the suspected time of infection [12,13]. However, the SOFA score is continuously measured only for patients in the intensive care unit (ICU), as in the case of the Glasgow coma scale.

For these reasons, although existing models have shown significant results, they cannot be used in the general ward especially when factors other than infection, such as surgery, may affect the SIRS criteria through inflammation or when the SOFA score cannot be measured. To overcome the limitations of indirect measurements of infection, direct measurements of blood culture could be helpful for defining infection. In the EHR data set, the results of a blood culture are recorded along with the reception time and the isolated species; therefore, clinicians can define the period of bacteremia so that the labeling of bacteremia only represents the infection. Once a clinician identifies the risk for patients, the clinician could investigate the source of infection and prescribe antibiotics.

Recently, the time series models based on the long short-term memory model [14] and the gated recurrent unit [15] have gained popularity due to their end-to-end modeling, ease of incorporating exogenous variables, and ability for extracting features [16]. The models use a time window to characterize the trend of features. Time series data such as vital signs or laboratory measurements could have different features whether the body temperature increases slowly or quickly. Moreover, if the model uses a longer time window, the model could imply a longer trend of data. With the ability to characterize the trend of time series data, previous studies showed significant performance using those models for predicting sepsis or acute kidney injury [8,17].

This paper presents a model based on a recurrent neural network (RNN) that continuously detects and predicts bacteremia for surgical in-patients in the general ward and the ICU. We compared the performance of this model with the traditional models used in sepsis detection (ie, SIRS criteria, SOFA score, and MEWS). To enhance the reader’s understanding, the paper also presents figures depicting continuous detection alongside vital signs and laboratory measurements.

## Methods

### Study Population

We retrospectively included all patients who had undergone general surgeries at Asan Medical Center (Seoul, South Korea) between October 2007 and December 2017. The following surgical procedures, coded by the International Classification of Diseases, Tenth Revision, Clinical Modification (ICD-10-CM), were included: lung lobectomy (32.0-32.4), gastrectomy (43.4-43.9), hepatectomy (50.0-50.4), and pancreaticoduodenectomy (51.5-51.7, 52.7). We excluded patients who did not undergo spirometry within 3 months before the surgery as well as those who received operations other than the previously mentioned types according to the manually written operation records (first e-table in Multimedia Appendix 1). For querying the EHRs, we used the Asan Biomedical Research Environment system at Asan Medical Center [18,19].

The data can be categorized into time-invariant and time-variant data depending on whether the data changed over the admission period. The time-invariant data included demographic data (ie, age, sex, height, weight, body mass index), underlying disease as coded with the ICD-10-CM code, type of operation, disease for operation, amount of transfusion during operation, spirometry (forced expiratory volume in 1 second [FEV_1_], forced vital capacity [FVC], FEV_1_/FVC, each with raw and predicted values), and the department of surgery. The time-variant data were vital signs (systolic blood pressure [sBP], diastolic blood pressure [dBP], pulse rate, respiratory rate, temperature) and laboratory data (groups as white blood cell [WBC], red blood cell, liver function, electrolyte, kidney function, arterial blood gas analysis, inflammation).

### Definition of Bacteremia

Bacteremia was defined as a laboratory-confirmed bloodstream infection that meets at least one of the following criteria [1]. First, the patients must have a recognized pathogen cultured from ≥1 blood specimen. Second, patients must have a fever (>38.0 °C) or hypotension (sBP<90 mmHg) in case of common skin contaminant (eg, diphtheroids, *Bacillus* species, *Propionibacterium* species, coagulase-negative staphylococci, or micrococci) that should be isolated from more than 2 blood cultures.

### Ground Truth for Bacteremia Periods

Two time points of bacteremia were present in the EHR data set: time ordered by a clinician and time of reception at the department of laboratory. Often there existed a time discrepancy between a clinician’s order and the actual sampling time. Therefore, the reception time (which was a little later than sampling time) was used as the ground truth time point of the bacteremia. In a previous study, bacteremia episodes identified in more than one blood culture within 24 hours were considered as a single episode [20]. In general, within the 24-hour period, vital signs are not readily stabilized even with the appropriate use of antibiotics. Thus, we defined the “bacteremia period” as 24 hours after the time of bacteremia and labeled it as the ground truth.

The prediction target at each point in time prior to the blood culture was a binary variable that was deemed positive if the bacteremia occurred within a predetermined time window. To determine the effect of the length of the time window on the detection ability of the model, we trained three different models (8, 16, and 24 hours prior) for predicting future time points.

### Models for Detecting and Predicting Bacteremia

All models function across the entire time period of admission. In the beginning of an admission period, there were not enough data to fill each time window. In these cases, only the existing data were used for predicting bacteremia. For example, to predict bacteremia 24 hours after admission, with a 96 hour (4 days) time window, only data from the first 24 hours of admission were used, and the vacant 72 hours of data, which was prior to the admission, were masked (Keras Masking layer). We tested time-variant variables with different time steps (1, 2, 4, 6, 8, 10, and 12 days) to find the optimal length of time steps that results in superior detection performance.

When constructing the model, different approaches were taken depending on the nature of the variables: time-variant variables were treated with an RNN-based model and time-invariant variables were treated with dense neural networks (first e-Figure in Multimedia Appendix 1). The outputs of both arms were concatenated, and the probability of bacteremia was calculated by a dense neural network. The code used to train and evaluate the model is available on GitHub [21].

We used the area under the receiver operating characteristic curve (AUROC), area under the precision-recall curve (AUPRC), sensitivity, specificity, and positive predictive value as comparative measures. The 95% CI was calculated by using a bootstrap approach in which we resampled the data at each time point 1000 times. All analyses were conducted in Python, version 3.7.5 (Python Software Foundation).

Details on data preprocessing, feature embedding, hyperparameter optimizing, ensemble of each batch for overcoming imbalance of data, and model testing are described in the method section of Multimedia Appendix 1.

### Significance of Features Analysis

The performance of the model changes when some variables are masked. This method is known as occlusion analysis, which is frequently applied in the field of image analysis [17,22]. We investigated the relative importance of the variables in our trained models through the occlusion analysis, in which variable groups were occluded one by one to determine their respective influence on the prediction of bacteremia. For example, if the occluded variables held higher importance in bacteremia detection, the resulting model would have a lower performance. Each group of variables was independently embedded with separate autoencoders, and the groups are described in the second e-Table in Multimedia Appendix 1.

## Results

### Baseline Characteristics

A total of 56,339 patients and 58,223 admission records were included when applying ICD-10-CM codes and spirometric results. After applying the exclusion criteria, 35,256 patients and 36,023 admission records were left in the final data set. The baseline demographic characteristics, operation types, and hours of operation are described in the third e-Table in Multimedia Appendix 1. The incidence of bacteremia was 1.9% (720/36,023 cases) and the period of bacteremia was 0.22% (1006/1,362,865 person-time). Furthermore, the annual incidence of bacteremia was rather stable during the study period, ranging from 1.6% (76/4484 cases) to 2.3% (45/1882 cases; second e-Figure in Multimedia Appendix 1).

The median time of bacteremia was 9 (IQR 2-18) days since admission, and the median time of postoperative bacteremia was 9 (IQR 6-15) days after surgery. If bacteremia occurred prior to surgery, patients underwent surgeries at a median of 10 (IQR 5-18) days after the occurrence of bacteremia (third e-Figure in Multimedia Appendix 1).

### Predicted Probability in Laboratory Sheets

**Figure 1:**
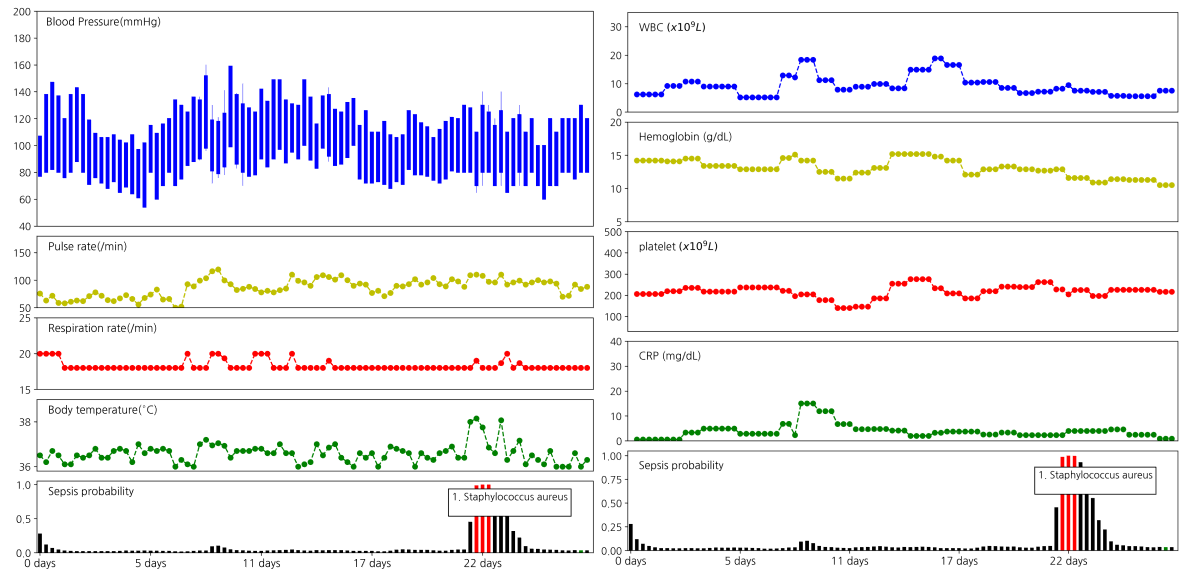
Patterns of the probability of bacteremia along with vital signs and laboratory data. Data from a 76-year-old female patient admitted for pancreatic cancer who underwent pylorus-preserving pancreaticoduodenectomy on hospital day 8. The graph in the bottom shows the probability of bacteremia at each time step. Red bars represent the actual period of bacteremia during which bacteria was isolated in the blood culture. The name of the pathogen is written in a small box. On hospital day 21, fever was noted and the probability of bacteremia was elevated. Lab data did not show a notable correlation with bacteremia probabilities. CRP: c-reactive protein; WBC: white blood cell.

Figure 1 depicts the predicted probability of bacteremia along with the vital signs and the laboratory data during hospital admission. As shown in Figure 1, the probability of bacteremia notably increased when the pulse rate and body temperature were elevated. In contrast, laboratory data such as WBC, hemoglobin, platelet, and c-reactive protein (CRP) did not show such changes in accordance with the increase in bacteremia probability (Figure 1). More examples of good, bad, and obscure prediction results are presented in the fourth e-Figure in Multimedia Appendix 1.

**Figure 2:**
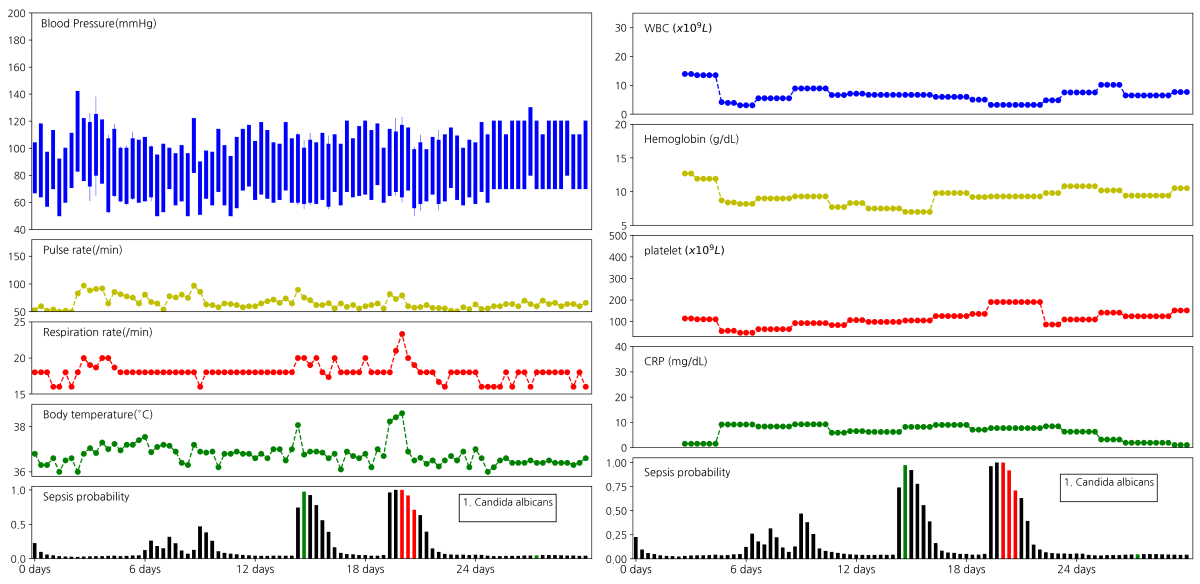
Time of negative blood culture could represent high likelihood of bacteremia. Data from a 77-year-old male patient admitted for intrahepatic duct stone. The lobectomy of the liver was carried out on hospital day 3. On hospital day 15, high fever was noted, and the blood culture was performed; however, no bacterial species were isolated. On hospital day 20, the second high fever was identified, and the blood culture was performed again. Candida Albicans was isolated, and the vital sign was subsequently stabilized. The green bar is the blood culture with no isolation. CRP: c-reactive protein; WBC: white blood cell.

Figure 2 shows the actual time of negative blood cultures as green bars. Even though the model was only trained based on the red bars (ie, bacteremia periods), the green bars indicate high probabilities for bacteremia. As the results of a blood culture could be false negative, the green bar might represent bacteremia at which clinicians should inspect patients and prescribe antibiotics. Other examples related to this figure are shown in the fourth e-Figure in Multimedia Appendix 1.

**Figure 3:**
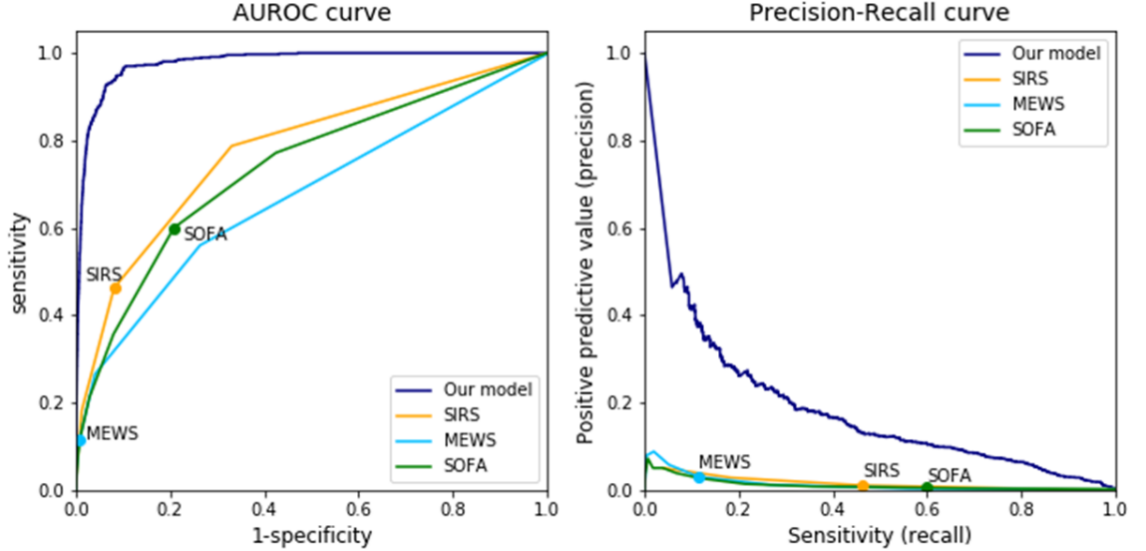
Receiver operating characteristics and precision-recall curve of the proposed model. The AUROC of the model was 97%, and the area under the precision-recall curve was 17%, which were higher compared with those of previous models. Each circle of previous criteria is the metric of the cut-off value of the models. AUROC: area under the receiver operating characteristic curve; MEWS: Modified Early Warning Score; SIRS: systemic inflammatory response syndrome; SOFA: Sequential Organ Failure Assessment.

Our model had an AUROC of 0.978 (95% CI 0.974-0.981) and an AUPRC of 0.17 (95% CI 0.147-0.203; Figure 3). The AUROC of previous models are as follows: SIRS 0.778 (95% CI 0.768-0.786), SOFA 0.738 (95% CI 0.728-0.748), and MEWS 0.673 (95% CI 0.662-0.682). The AUPRC of previous models are as follows: SIRS 0.011 (95% CI 0.010-0.013), SOFA 0.010 (95% CI 0.009-0.011), and MEWS 0.010 (95% CI 0.008-0.011).

Table 1 shows the trend of sensitivity and specificity of the proposed model according to different thresholds, along with the performances of other models (ie, SIRS, SOFA, and MEWS). The positive predicted value was relatively low because of the low incidence of bacteremia in our data set. Overall, our model showed superior performance to the SIRS criteria [23], SOFA score [6], and MEWS score [24] in terms of both AUROC and AUPRC for detecting bacteremia.

**Table 1:** Performance of the model compared with previous criteria.

Model and threshold		Sensitivity	Specificity	PPV^a^
**Recurrent neural network model**				
	0.1	0.94	0.92	0.023
	0.2	0.88	0.95	0.034
	0.3	0.86	0.96	0.044
	0.4	0.83	0.97	0.054
	0.5	0.79	0.98	0.065
	0.6	0.72	0.98	0.079
	0.7	0.65	0.99	0.099
	0.8	0.53	0.99	0.122
	0.9	0.41	0.99	0.165
SIRS^b^ criteria (>2 score)		0.46	0.92	0.011
SOFA^c^ score (>2 increase from baseline)		0.60	0.79	0.006
MEWS^d^ score (>4 score)		0.12	0.99	0.031

^a^PPV: positive predictive value.

^b^SIRS: systemic inflammatory response syndrome.

^c^SOFA: Sequential Organ Failure Assessment.

^d^MEWS: Modified Early Warning Score.

### Various Experiments for Bacteremia Prediction

We developed models predicting 8, 16, and 24 hours before the event of bacteremia (Table 2). Both the AUROC and AUPRC values decreased as the time to predict bacteremia increased from 8 to 24 hours (AUROC 0.963 to 0.929; AUPRC 0.176 to 0.154).

When we made the model using various time step lengths (1, 2, 4, 6, 8, 10, and 12 days), the AUROC values did not show notable decreases (0.977 to 0.971) as the time windows increased from 1 to 12 days; in contrast, the AUPRC value increased from 0.139 to 0.174 (Table 2). Performance for predicting bacteremia events 8, 16, and 24 hours prior to the event are shown. Different time steps and performances in our data set are shown. The time steps indicate the length of time period used in the RNN model to predict bacteremia.

**Table 2:** Model performance for predicting bacteremia according to forecasting time to event and time steps of the recurrent neural network model.

Variables		AUROC^a^	AUPRC^b^
**Prior time to event (hour)**			
	0 (at event)	0.98	0.17
	8 prior	0.96	0.18
	16 prior	0.95	0.17
	24 prior	0.93	0.15
**Time steps (days)**			
	1	0.98	0.14
	2	0.98	0.15
	4	0.98	0.15
	6	0.98	0.15
	8	0.97	0.16
	10	0.97	0.174
	12	0.98	0.165

^a^AUROC: area under the receiver operating characteristic curve.

^b^AUPRC: area under the precision-recall curve.

### Relative Importance of Variables

Table 3 shows the results of the occlusion analysis. We found that occlusion of vital signs resulted in the largest decrease in both the AUROC and AUPRC values, followed by kidney-related values and WBC. In contrast, the occlusion of time-invariant data, which were reported to be important in predicting postoperative complications [25-27], showed little effects in decreasing AUROC or AUPRC. For comparison with previous studies that only used time-invariant data [25-27], we also trained a model only with time-invariant data; as a result, we observed that this model had a similar performance with those in previous studies (AUROC 0.84, AUPRC 0.15; fourth e-Table in Multimedia Appendix 1). Vital signs and lab data were more important than time-invariant data, even though the latter could also somewhat assess the risk of bacteremia. Particularly, body temperature was the most important vital sign in detecting bacteremia, followed by dBP and pulse rate (fifth e-Table in Multimedia Appendix 1).

The model performance was described as when the important variables were occluded.

**Table 3:** Detecting performance of the proposed model in occlusion analysis.

Methods		AUROC^a^	AUPRC^b^
Original model		0.98	0.17
**Occluding method**			
	Occluding vital sign	0.85	0.05
	Occluding kidney-related values	0.95	0.06
	Occluding WBC^c^	0.96	0.07
	Occluding electrolyte	0.96	0.10
	Occluding RBC^d^-related lab	0.97	0.11
	Occluding ABGA^e^	0.97	0.12
	Occluding inflammatory markers	0.98	0.14
	Occluding time-invariant data	0.97	0.14
	Occluding liver function test	0.98	0.15

^a^AUROC: area under the receiver operating characteristic curve.

^b^AUPRC: area under the precision-recall curve.

^c^WBC: white blood cell.

^d^RBC: red blood cell.

^e^ABGA: arterial blood gas analysis.

## Discussion

### Summary of the Principal Finding

By using the deep learning method, we devised a model that generates a real-time probability of detecting and predicting bacteremia. The proposed model had an AUROC of 0.978 in detecting bacteremia every 8 hours, which is a notably superior performance compared with other existing criteria [2,5,6,24]. In predicting bacteremia 24 hours in advance, the model showed a relatively lower performance (AUROC 0.929) than the detecting model. Occlusion analysis showed that vital signs were the most important variables in bacteremia detection. Confirming our expectation that patterns of time-variant variables such as vital signs could be used to characterize the risk type of a patient, the model using long time steps showed more accurate results (time steps: 10 days vs 1 days, AUPRC: 0.174 vs 0.139).

This study is also important for the continuous monitoring of bacteremia so that clinicians can get advice on the risk of uncontrolled infection. Other studies on predicting sepsis rely on assessments about the clinical state (based on the SOFA or SIRS criteria) [8,9,20]. However, if the outcome label included systemic inflammations as well as infections, the probability results would be difficult for interpretation by clinicians [2]. To overcome the unclear labeling problem, we used the direct results from the blood culture and suggested the “bacteremia period,” which can be used as an indication of infection. Since the model derives the results solely from the blood cultures, the resulting predicted probabilities directly indicate the infection so that physicians can get advice when they search for the source of infection, start new antibiotics, or monitor the appropriate response to antibiotics.

### Importance of Variables in the Deep Learning Model

Deep neural networks are often questioned for being nontransparent and because the basis of the prediction results is hard to explain [28]. By depicting the probability with vital signs and major laboratory findings, we were able to determine whether the prediction results were proper (fourth e-Figure in Multimedia Appendix 1). Specifically, we observed that the bacteremia probability was elevated in accordance with increases in body temperature, respiratory rate, and pulse rate, whereas laboratory data such as WBC and CRP did not show notable correlations with the bacteremia probability.

To explain what variables drive the model, we used occlusion analysis, a method used in image analyses [28]. If the model is driven by an important location within the image, the result must not be changed after occluding the surrounding of the image [22]. We found that the most important variable of our model was the vital sign, followed by kidney-related values and WBC. The result resembles the SIRS criteria, which consists of three vital sign categories [2], and supports our expectation that our model would focus on relevant variables for predicting bacteremia. Underlying diseases are also known as important predictors of postoperative complications [25-27]. However, time-invariant variables such as the underlying diseases and the type of surgery did not have significant effects in the detection of bacteremia. In clinical practice, patients with a high likelihood of postoperative complications are not always suspected of having an infection, unless they show features of infection such as fever. As previous risk factors suggest that only high-risk patients acquired infection during the whole admission period, the detection of bacteremia should be based on clinical clues such as vital signs or laboratory data. This is in line with the routine practice of clinicians suspecting infection based on vital signs and clinical features rather than underlying disease.

### Validation of Predicted Probability Compared With Medical Chart

Blood cultures are usually performed for diagnosing infections or monitoring the bacteremia, and some negative blood cultures could be false negatives [1]. Investigating negative blood cultures (green bars in the fourth e-Figure in Multimedia Appendix 1), the predicted probability of the green bar is high among negative cases (Figure 2). However, a green bar following 2 days after a positive blood culture showed a lower probability of bacteremia (4.2.2 e-Figure in Multimedia Appendix 1). Although the negative blood culture was not trained in our model, the probability at the green bar represents the time of the blood culture following a clinician’s suspicion. Usually, the probability for bacteremia is high at the green bar, meaning that both clinicians and our model suspected high risk of infection at similar time points. In other words, our model is not just trained for the labeling but also trained against general features of bacteremia.

Additionally, we examined the performance of our model using different time steps. We assumed that additional information exists when there are spikes in body temperature or lab data, or when the data exhibits recognizable patterns throughout the time. Using the RNN-based model, such information can be considered in the hidden state and be used to predict bacteremia. When we increased the time steps per bacteremia prediction, the performance of the model in bacteremia detection was increased, indicating that the model was able to further learn the patterns of vital signs and lab data. For example, when the changes in value were not steep, the predicted probability was relatively low despite the elevated pulse rate and body temperature (4.1.3 e-Figure in Multimedia Appendix 1). These patterns of vital signs and lab data could also be used for differentiating different species of bacteremia if they have distinct disease patterns.

### Limitations

Our study has the following limitations. First, we used the outcome defined by positive blood culture. In the Prehospital Antibiotics Against Sepsis Trial, only 42.6% of the cases were culture-positive sepsis [29]. Therefore, this labeling may have affected our model and the results. However, because we assumed that the vital signs and laboratory results are similar between culture-positive sepsis and culture-negative sepsis, the model could predict higher probability even when the blood culture produced negative results. Further prospective study is needed to validate the proper prediction about culture-negative sepsis. Second, our model was trained on data from a single tertiary hospital in Korea and may, thus, have limited generalizability. Nevertheless, our data set does not seem to significantly deviate from the country-wide data, as the incidence rates of bacteremia in our data set and the general Korean cohort data were 1.9% and 2.2%, respectively [30]. As our data set included all consecutive patients who underwent surgeries, it could represent the global population of surgical patients undergoing major upper abdominal surgery and thoracic surgery. In addition, as our model used the data set in a retrospective manner, a prospective study is needed to determine whether our proposed model confers real-time values in helping clinicians predict bacteremia at an earlier stage.

### Conclusions

In conclusion, we have applied the deep learning algorithm to develop a model for detecting and predicting bacteremia with in-hospital data. Our model may help clinicians to make appropriate decisions regarding early responses to bacteremia. In the future, clinicians may be able to improve the clinical outcomes of patients with bacteremia using this algorithm in the EHR system.

## Appendix

Multimedia Appendix 1 Supplementary material.

## Abbreviations

AUPRC: area under the precision-recall curve
AUROC: area under the receiver operating characteristic curve
CRP: c-reactive protein
dBP: diastolic blood pressure
EHR: electronic health record
FEV1: forced expiratory volume in 1 second
FVC: forced vital capacity
ICD-9: International Classification of Diseases, Ninth Revision
ICD-10-CM: International Classification of Diseases, Tenth Revision, Clinical Modification
ICU: intensive care unit
MEWS: Modified Early Warning Score
RNN: recurrent neural network
sBP: systolic blood pressure
SIRS: systemic inflammatory response syndrome
SOFA: Sequential Organ Failure Assessment
WBC: white blood cell
