# Supplementary material for: Detection of Bacteremia in Surgical In-Patients Using Recurrent Neural Network Based on Time Series Records: Development and Validation Study
Source: J Med Internet Res. 2020 Aug 4;22(8):e19512. doi: 10.2196/19512 (PMC7435626; doi:10.2196/19512)
Supplement: Multimedia Appendix 1 [file jmir_v22i8e19512_app1.docx]

**Supplement for: Detection of bacteremia in surgical in-patients using recurrent neural network based on time-series electronic health record**

Hyung Jun Park^1^, Dae Yon Jung^2^, Wonjun Ji^1^, Chang-Min Choi^1,3^

Affiliations: ^1^Department of Pulmonary and Critical Care Medicine, Asan Medical Center, University of Ulsan College of Medicine, Seoul, Korea, ^2^Big Data & AI Lab, Hana Institute of Technology, Hana TI, Seoul, Korea; ^3^Department of Oncology, Asan Medical Center, University of Ulsan College of Medicine, Seoul, South Korea

**Table of Contents:**

Section 1. Supplement method …….……………………………..………………………….. 3

Preprocessing ……………………………………………...………………………... 3

Embedding modules ………………………………………………………………… 4

Recurrent neural network ………………………………………………………….... 5

Ensemble of mini batch ……………………………………………………………... 5

Training and hyperparameter …………………………………………..…..………... 5

Section 2. Supplement Tables …………………………………………….………..………… 7

Supplement e-Table 1… …………………………………………….…………….… 8

Supplement e-Table 2 ………………………………………….…………….…… 9

Supplement e-Table 3 ………………………………………….……………........ 10

Supplement e-Table 4 ………………………………………….………………… 11

Supplement e-Table 5 …………………………………………….……………… 12

Supplement e-Table 6 ……………………………………………………………. 13

Section 3. Supplement Figures ……………………………………………..…………….… 14

Supplement e-Figure 1… …………………………………………….…….……… 14

Supplement e-Figure 2 …………………………………………….…….………. 15

Supplement e-Figure 3 …………………………………………….…………….. 16

Supplement e-Figure 4 …………………………………………….……..….…… 18

Supplement e-Figure 4 …………………………………………….……..………. 24

Section 4. References …………………………….………………………………………… 27

**Section 1. Supplement method**

**Preprocessing** After data curation, we matched the ICD-10-CM code with the actual operation name. There were several cases with the mismatch: for instance, a patient may have the operation (ICD-9-CM) code for partial hepatectomy, and actually underwent liver transplantation; in another case, the operation code may indicate partial gastrectomy but the patient actually underwent endoscopic procedures and discharged after two days after the operation, which is not feasible in other patients with gastrectomy. For this reason, we re-sorted the operation names and diagnosis names by using the Regular Expression in the Python code.[1] We regarded that the operation name written in the operation record was more appropriate than the ICD-9-CM code in the discharge record. Thus, we excluded the patients with mismatches between their operation names and ICD-9-CM codes. The excluded names of the operations are described in Supplement Table 1. In the cases of patients undergoing more than one operation during the admission period, we only included the first operation. The comorbidity diseases were acquired as ICD-10 codes, and the codes are described in Supplement Table 6. These codes, such as diagnostic or operation names, were encoded as binary features.

Time-variant data were categorized into vital signs and laboratory data. In the general ward of Asan Medical Center, vital signs are usually checked at least three times a day, and the laboratory data are checked once a day. Thus, the base time unit for data sampling was set as 8 hours. In this study, vital sign was recorded as spot-check data and not as continuous data. If the vital signs or lab data were checked more than once within 8 hours, we summarized the data as max, min, mean, count, and standard deviation. In order to include the feature variance with time, the difference between the current and the last value of the mean was included as a feature, similar to the representation method used in a previous study. [2] Because the patient measurements were recorded irregularly, not all 8-hour time-steps in a given day had new data. If a patient did not have a measurement in a given time-step, the missing measurement was filled in using carry-forward imputation by applying the patient’s last measured value to the following hour.[3] If the data were not available at the early day of the admission, we imputed the reference mean value of this whole dataset that can represent the value within the normal range. Before checking the vital signs or laboratory data, physicians usually decided on the diagnosis based on the normal value until abnormal vital signs or laboratory data were noticed. After processing the data with these series of approaches explained above, we can make predictions at every time step.

All numerical features were capped at the 1^st^ and 99^th^ percentiles in order to prevent the normalization form being dominated by a potentially large value caused by human entry error while preserving most of the information from the feature set.[2] Skewed data were transformed with Box-Cox transformation[4]: left-skewed data were transformed into log-scale, and right-skewed data were transformed after flipping left to right (max value +1 – value). Data before and after the transformation are shown in Supplement Figure 1.

**Embedding modules.** The embedding transforms the high-dimensional and sparse input features into lower-dimensional and continuous representations in order to facilitate subsequent prediction. [2] We used a stacked autoencoder for embedding that the model composed of the symmetric deep neural network as an encoder and a decoder. We used an L_2_ regularization on the embedding, and the variables have been previously transformed using Box-Cox transformation so that the model can focus on the most salient features.

We grouped the input variables that have similar semantic values. The components of the groups are described in Table 2**.** The variables of each group were trained in separate autoencoders. The best performing layer was achieved with six layers of auto-encoder (three encoders, three decoders), and the dimension of the code was 12. This embedding showed superior performance than the raw input model. We used the code layer of the autoencoder as the time-invariant input in our model.

**Recurrent neural network.** Recurrent neural network (RNN) is a type of artificial neural network that is commonly used in forecasting time-series data.[5] RNN runs sequentially over the in-hospital data entries and is able to recognize the patterns of the input data. We chose the architecture from our model after training and testing two widely used RNN architectures: the Long Short-Term Memory (LSTM)[6] and Gated Recurrent Units (GRU)[7]. The two trained models showed similar performance; however, GRU was selected as our final RNN architecture as it fitted faster than LSTM while showed similar performance of predicting bacteremia.

**Ensemble of mini-batch** We investigated the use of ensemble to account for the class imbalance. Each mini-batch contained all of the positive samples, 20% of the negative samples from patients who had bacteremia during the admission period, and 10% of the negative samples from patients who did not have bacteremia. Thus, each batch had 17% of positive samples, and this percentage is much larger than the raw incidences of bacteremia. (1.9% patients-wise, 0.22% time-wise)

**Training and hyperparameters.** All variables were initialized via the normalized initialization (Xavier)[8], the default initializer of Keras, and trained using the Adam optimization. [9] The best validation results were achieved using an initial learning rate of 0.003, with a random batch size of 30000 to 40000. The time window of the model was 30 steps (10 days).

In the RNN, we tested various dimensionality units of the output space: 8, 16, 32, 64, and 128. In the Dense neural network of time-independent variables, the dimensionalities of the output layer were also 8, 16, 32, 64, and 128. Dropout was used in the outputs of the dense_2 layer, RNN layer, and the dense_final layer. (Supplement Figure 1) The dropout ratio was 0.5. The overall loss function in our model was the sum of the binary cross-entropy from the bacteremia predictions.

**Evaluation.** The final dataset was randomly split into the training (60%), validation (20%), test (20%) sets. The split was based on the patients so that the samples from the same patient cannot be simultaneously included in the training, validation, or test sets.

**Previous criteria for sepsis**. SOFA score was calculated according to the Sepsis-3 criteria.[10] As PaO_2_ and Glasgow coma scale are not routinely checked in the general ward, the SOFA score was calculated based on other categories (e.g., platelets, bilirubin, mean BP, creatinine). We assumed that the baseline SOFA score was zero, as recommended in the sepsis-3 consensus definition.[11]

The SIRS criteria are based on the heart rate, respiratory rate, body temperatures, and white blood cell count. Based on the criteria, we made each score at each 8 hours time-step. The MEWS score was based on the respiratory rate, heart rate, systolic BP, urine output, temperature, and neurological sign. Urine output and neurological sign are not usually checked in general wards; therefore, we used labels other than urine output and neurologic sign. The detailed process code is available at our github (<https://github.com/podkd87/bacteremia>).

**Section 2. Supplement Tables**

Supplement e-Table 1. Names of the actual operation that were mismatched with the ICD-9-CM code.

| **Thoracic surgery** |
| --- |
| Ivor Lewis operation, pulmonary thromboembolectomy, Mckeown operation, pleuropneumonectomy, esophago-jejunostomy, portal vein resection, pericardiostomy, open heart surgery, esophageal interposition, wound revision, decortication, Maze operation, thoracic duct ligation, fistulectomy, conduit take down, veno-veno ECMO, esophagectomy, primary repair, postop bleeding, bleeding control. |
| **Liver surgery** |
| Laparoscopic-distal pancreatectomy, total pancreatectomy, pancreatico-splenectomy, laparoscopic distal splenectomy, distal pancreatico-splenectomy, donor hepatectomy, liver transplantation, laparoscopic cystectomy, cyst excision, nephrectomy, adrenalectomy, open biopsy, irrigation, bleeding control, exploratory laparotomy, wound revision, adhesiolysis. |
| **Gastric surgery** |
| Chest tube insertion, laparoscopic gastric conduit formation, gastrojejunostomy, bariatric surgery, wound revision, gastroduodenostomy. |

ECMO, extracorporeal membrane oxygenation

There is a discrepancy between ICD-9-CM code for surgeries and their actual name. We sorted the actual names of the surgeries by a rule-based method using the Regular Expression. [1]

Supplement e-Table 2. Groups of input variables for embedding in auto-encoder.

| Time-invariant variables | | |
| --- | --- | --- |
| Operation  (binary) | Lung | Wedge resection, VATS, lung lobectomy, lung segmentectomy, lung metastasectomy, mediastinal lymph node dissection, bilobectomy, exploration thoracotomy, sleeve lobectomy, |
|  | Liver | (laparoscopic) left lobectomy, (laparoscopic) right posterior segmentectomy, extended right lobectomy, (laparoscopic) right anterior resection, partial hepatectomy, right lobectomy, central bisegmentectomy, extended left hepatectomy, laparoscopic hepatectomy (other), (laparoscopic) pancreaticoduodenectomy, bile duct resection, (open or laparoscopic) cholecystectomy, (laparoscopic) extended cholecystectomy |
|  | Stomach | (laparoscopic) gastrectomy, (laparoscopic) total gastrectomy, (laparoscopic) pylorus preserve gastrectomy, (laparoscopic) distal gastrectomy, (laparoscopic) wedge resection |
| Diagnosis  (binary) | Lung | Lung cancer, metastasis to lung, solitary pulmonary nodule, lung ground-glass opacity, pneumothorax, bronchiectasis, fungal ball, tuberculosis, congenital pulmonary airway malformation, lung infection |
|  | Liver | Klatskin tumor, intrahepatic duct stone, acute cholecystitis, chronic cholecystitis, metastasis to liver, hepatocellular carcinoma, hepatic mass, gallbladder cancer, biliary infection, gall bladder stone, gall bladder polyp, common-bile duct cancer, cholangiocellular carcinoma, Ampulla of vater cancer, pancreatic cancer, duodenal cancer |
|  | Stomach | gastric cancer, other mass in stomach |
| Underlying disease  (binary) | Charlson comorbidity index disease and some pulmonary disease | Coronary artery disease, congestive heart failure, chronic obstructive pulmonary disease, asthma, bronchiectasis, interstitial lung disease, lung abscess, cystic fibrosis, mild liver disease, moderate to severe liver disease, mild renal disease, moderate to severe renal disease, uncomplicated diabetes mellitus, complicated diabetes mellitus, dementia, cerebrovascular accident, peripheral vascular disease, peptic ulcer disease, AIDS, connective tissue disease, SLE, rheumatoid arthritis, scleroderma, Sjogren disease, psoriatic arthritis |
| Transfusion  (numerical) |  | Fresh Frozen Plasma, Red blood cell (RBC), platelet concentrate (PC), RBC-leukoreduced, RBC-leukocyte filtered, Cryoprecipitate, PC-leukocyte filtered (Each with amount of order and discharge) |
| Time-variant variables | | |
| Vital Sign | Systolic BP, diastolic BP, pulse rate, respiratory rate, body temperature | |
| RBC related | Hematocrit, RBC, hemoglobin, MCHC, MCV, MCH, RDW, MPV, PDW | |
| WBC related | WBC, neutrophil, lymphocyte, ANC, monocyte, eosinophil, basophil, immature granulocyte | |
| Liver function related | AST, ALT, bilirubin, ALP, cholesterol, PT, aPTT, Albumin, Protein | |
| Electrolyte related | Calcium, Phosphorus, Potassium, Sodium, Chloride, Magnesium | |
| Kidney related | eGFR, Creatinine, BUN, Uric acid | |
| ABGA related | pH, pO2, Saturation, pCO2, total CO2, bicarbonate, base excess, lactic acid, glucose | |
| Inflammation | CRP, amylase | |

ABGA: arterial blood gas analysis; AIDS: acquired immunodeficiency syndrome; ALT: alanine aminotransferase; ALP: alkaline phosphatase; aPTT: activated partial thromboplastin time; AST: aspartate aminotransferase; BP: blood pressure; eGFR: estimated glomerular filtration rate; MCH: mean corpuscular hemoglobin; MCHC: mean corpuscular hemoglobin concentration; MCV: mean corpuscle volume; MPV: mean platelet volume; PDW: platelet distribution width; PT: prothrombin time; RDW: red cell distribution width; SLE: systemic lupus erythromatosus; VATS: video-assisted thoracoscopic surgery; WBC: white blood cell.

Supplement e-Table 3. Baseline characteristics of study subjects.

|  | | Mean ± SD or number, (%) |
| --- | --- | --- |
| Age | | 60.4 ± 11.1 |
| Male sex | | 24780 (62.1) |
| Height(cm) | | 162.8 ± 8.4 |
| Weight(kg) | | 63.5 ± 10.7 |
| Operation time(hour) | | 3.14 ± 2.02 |
| Anesthesia time(hour) | | 3.57 ± 2.14 |
| **Spirometry** | |  |
|  | Measured FVC | 3.5 ± 0.86 |
|  | Predicted FVC (%) | 89.3 ± 13.6 |
|  | Measured FEV_1_ | 2.6 ± 0.7 |
|  | Predicted FEV_1_ (%) | 90.1% ± 15.6 |
| **Comorbidity** | |  |
|  | Cancer | 23626 (59.2) |
|  | Liver cirrhosis | 5315 (13.3) |
|  | Diabetes | 3453 (9.9) |
|  | Ischaemic heart disease | 402 (1.0) |
|  | Cerebral artery disease | 34 (0.1) |
|  | COPD | 340 (0.9) |
|  | Chronic kidney disease | 251 (0.8) |
|  | Heart failure | 35 (0.1) |
|  | ILD | 207 (0.5) |
|  | bronchiectasis | 180 (0.5) |
| **Operation** | |  |
|  | Lung lobectomy | 7347 (18.4) |
|  | Hepatectomy | 19109 (47.8) |
|  | Pancreaticoduodenectomy | 6190 (15.5) |
|  | Stomach-related (gastrectomy) | 9910 (24.8) |
|  | Length of hospital stay in days  (median, 25-75 percentile) | 10 (7-17) |

COPD: chronic obstructive pulmonary disease; FEV1: forced expiratory volume in 1 second; FVC: forced vital capacity; ILD: interstitial lung disease; SD: standard deviation.

Supplement e-Table 4. Models using only time-invariant variables

|  | AUROC | AUPRC |
| --- | --- | --- |
| Logistic Regression | 0.50 | 0.03 |
| Random Forest | 0.69 | 0.38 |
| Gradient Boosting model | 0.69 | 0.36 |
| Dense neural network | 0.84 | 0.15 |

The occlusion analysis showed that demographics, type of operations, and spirometric results have relatively low importance; however, by using only these parameters, bacteremia can be predicted with an AUROC of 0.84 and an AUPRC of 0.15, which are similar to value from previous studies.[12,13]

Supplement e-Table 5. Subanalysis of the detection performance among vital signs by occlusion analysis

|  | | AUROC | AUPRC |
| --- | --- | --- | --- |
|  | Original model | 0.98 | 0.17 |
| **Occluding method** | | | |
|  | Occluding whole vital sign | 0.85 | 0.05 |
|  | Occluding body temperature | 0.93 | 0.11 |
|  | Occluding diastolic pressure | 0.96 | 0.12 |
|  | Occluding pulse rate | 0.97 | 0.16 |
|  | Occluding respiratory rate | 0.98 | 0.17 |
|  | Occluding systolic pressure | 0.98 | 0.17 |

Supplement e-Table 6. ICD-10 codes for comorbidities.

| Name of disease | | ICD-10 code (first 3 digits) |
| --- | --- | --- |
| **Heart disease** | |  |
|  | Ischemic heart disease | I25- |
|  | Congestive heart disease | I50- |
| **Pulmonary disease** | |  |
|  | COPD | J44 |
|  | Asthma | J45 |
|  | bronchiectasis | J47, Q33 |
|  | Interstitial lung disease | J84 |
|  | Lung abscess | J85 |
|  | Cystic fibrosis | E84 |
| **Liver disease** | |  |
|  | Mild liver disease (except for code in severe liver disease) | K70, K71, K73 K74, B18 |
|  | Moderate to severe liver disease | K746W1, K7031, K7461, K7462, K746W3, K7011, K711, K7110, K7111, K711A, K7151, K715A, K716W1, K716W2 |
| **Renal disease** | |  |
|  | Mild renal disease | N18, N181, N182, N183 |
|  | Moderate to severe renal disease | N180W1, N180W2, N180W3, N180W4, N180W5, N180W6, N180W7, N184, N185, N188, N188A, N188B, N189, N189W1, N189W2, N189W3 |
| **Diabetes mellitus (DM)** | |  |
|  | uncomplicated DM | E10, E100, E1000, E1001, E1003, E1008, E101, E1010, E1018, E102, E1020, E11, E111, E1110, E1111, E1112, E1118, E111W1, E13, E1363, E139, E139W1, E139W2, E139W3, E139W4, E14, E14W1, E149, |
|  | complicated DM  (except code for uncomplicated DM) | E10-E14 |
| **Neurologic disease** | |  |
|  | Dementia | F01, F02, F03 |
|  | Cerebro-vascular accident | I61, I64 |
| AIDS | | B20, B21, B22., B23 |
| Cancer | | Including C code |

AIDS: acquired immunodeficiency syndrome; COPD: chronic obstructive pulmonary disease; DM: diabetes mellitus.

**Section 3. Supplement Figures.**

Supplement e-Figure 1. Architecture of the proposed model.


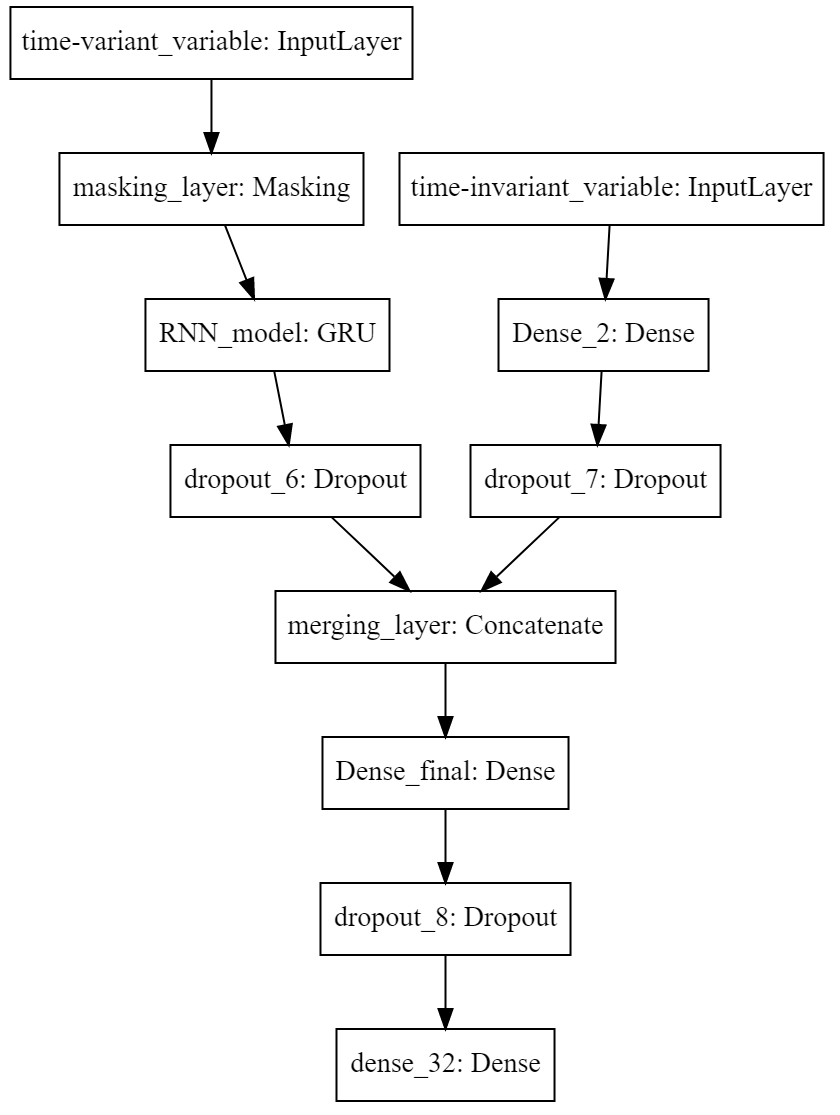


Two different types of data were treated with different networks: the RNN model was trained with the time-variant variables, and a dense layer was trained with the time-invariant variables. The outputs from these two networks were concatenated, and the dense layers were trained to produce the probability of bacteremia.

Supplement e-Figure 2. Annual incidence of bacteremia from 2008 to 2017.


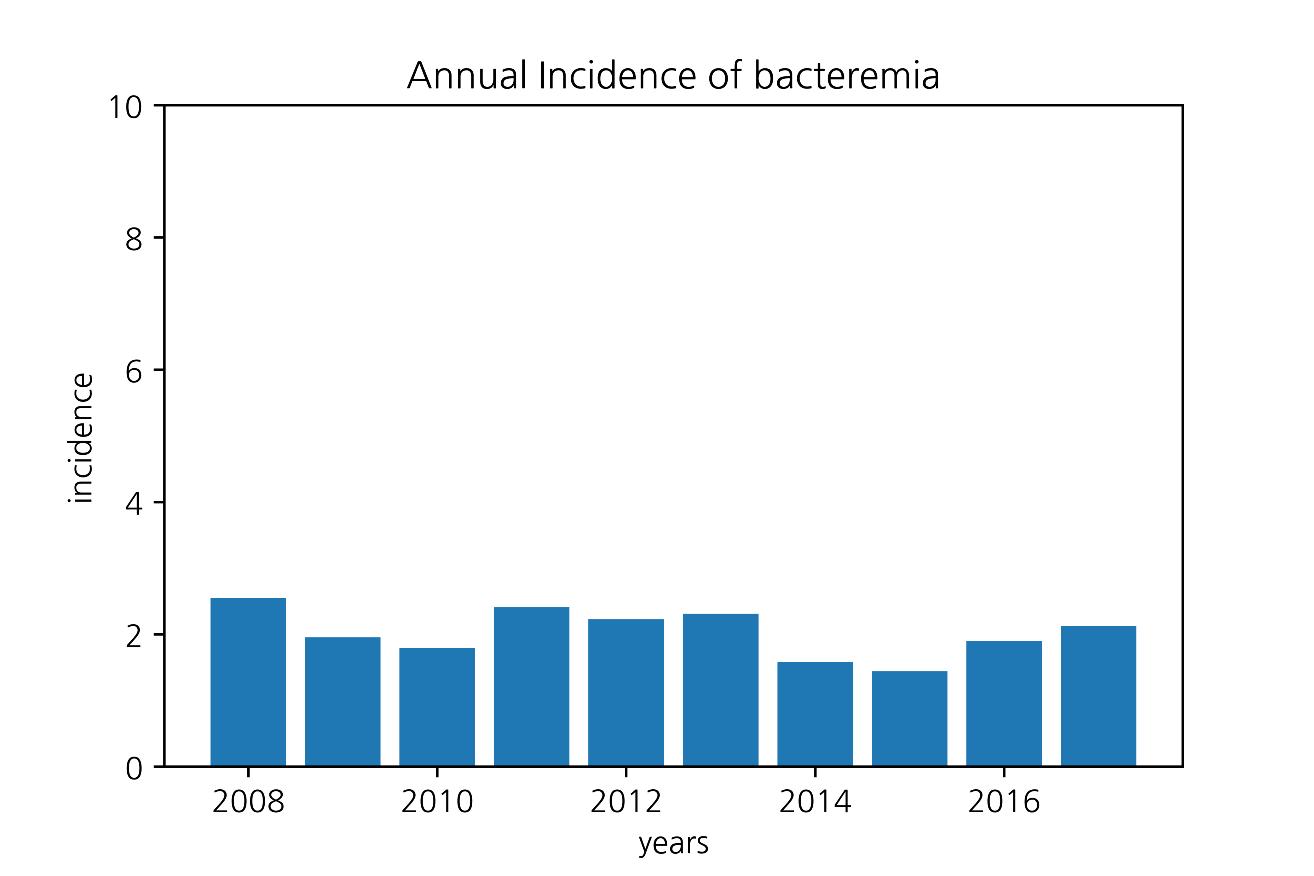


In 2007, only 35 patients were enrolled, and the incidence rate was 11.4%. The EHR system was established at the end of 2007, and thus only small amounts of patients were enrolled in that year. Therefore, we compared the number of enrollment in the years after 2007, which remained quite stable throughout the period.

Supplement e-Figure 3. Onset of bacteremia during admission.

(A)


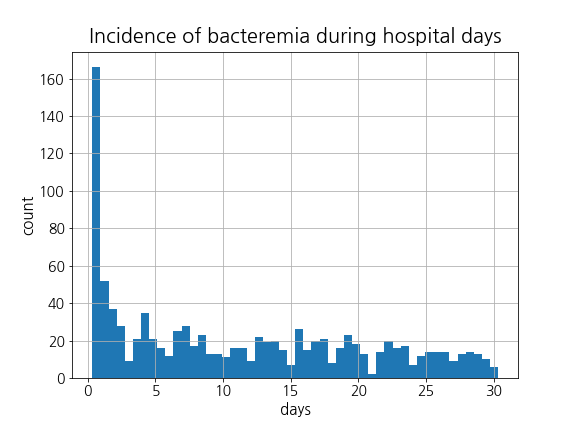


(B)


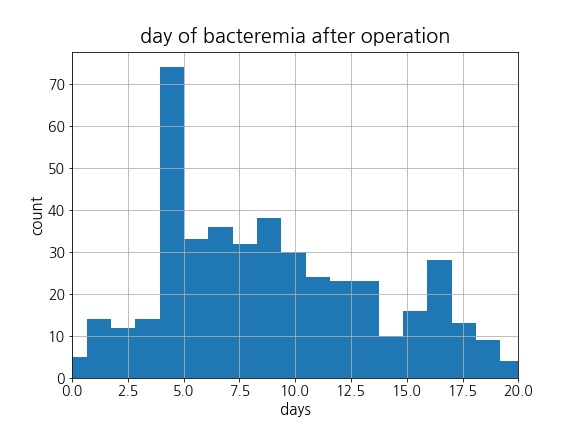


(C)


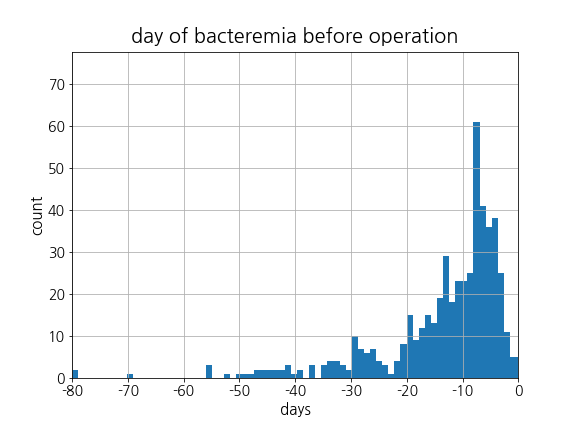


The day of bacteremia during the admission period. Figure (A) shows the date of bacteremia in whole hospital days. Figure (B) shows the date of bacteremia after the operation. Figure (C) shows the date of bacteremia before the operation.

Supplement e-Figure 4. Several cases of prediction.

4.1 Good prediction cases

4.1.1 Good prediction case 1


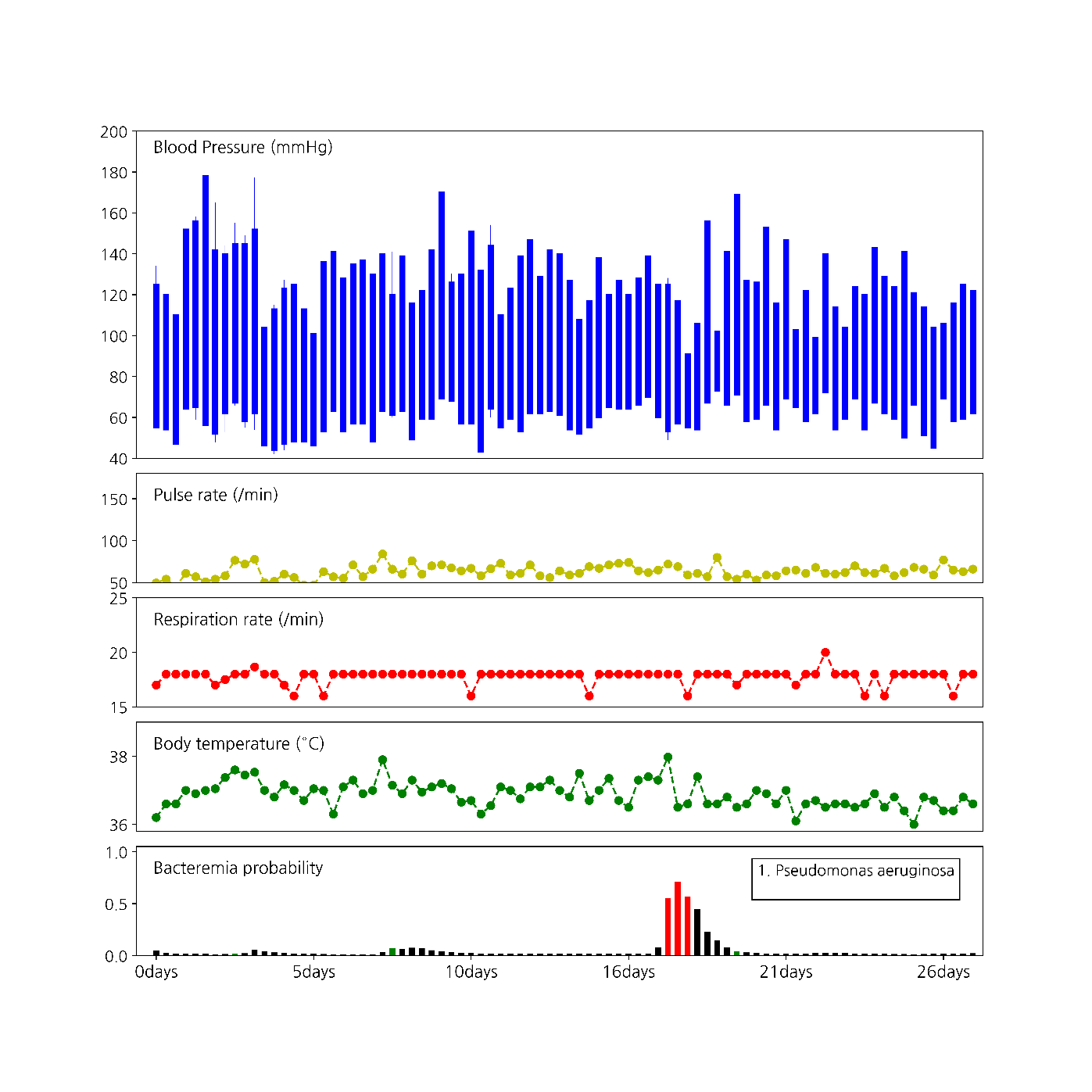

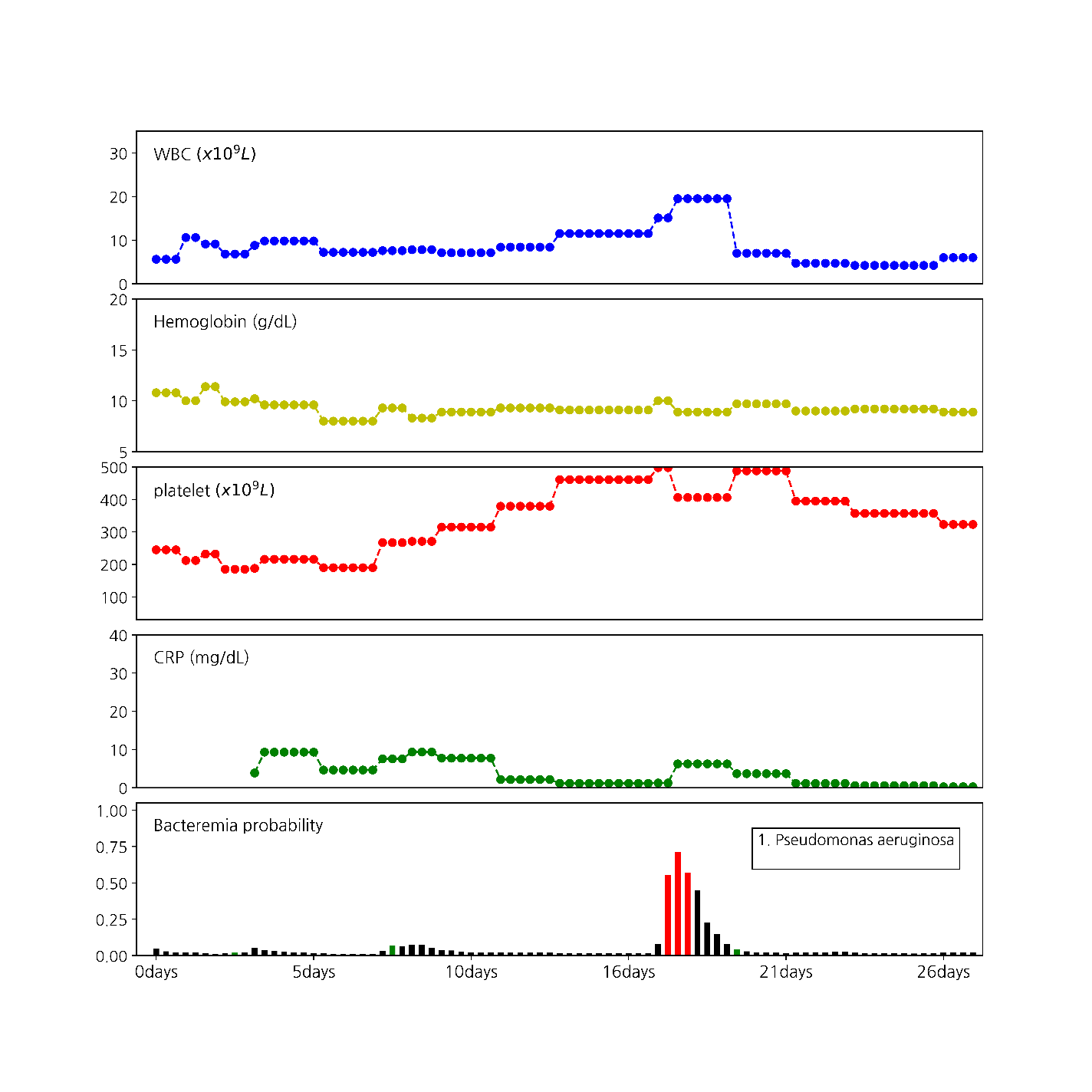


Data from a 73-year-old male patient admitted for gastric cancer. The operation was partial gastrectomy performed on hospital day 1. Even though the temperature had been slightly elevating in the first 16 hospital days, the predicted probability was elevated only when the blood culture was positive.

4.1.2 Good prediction case 2


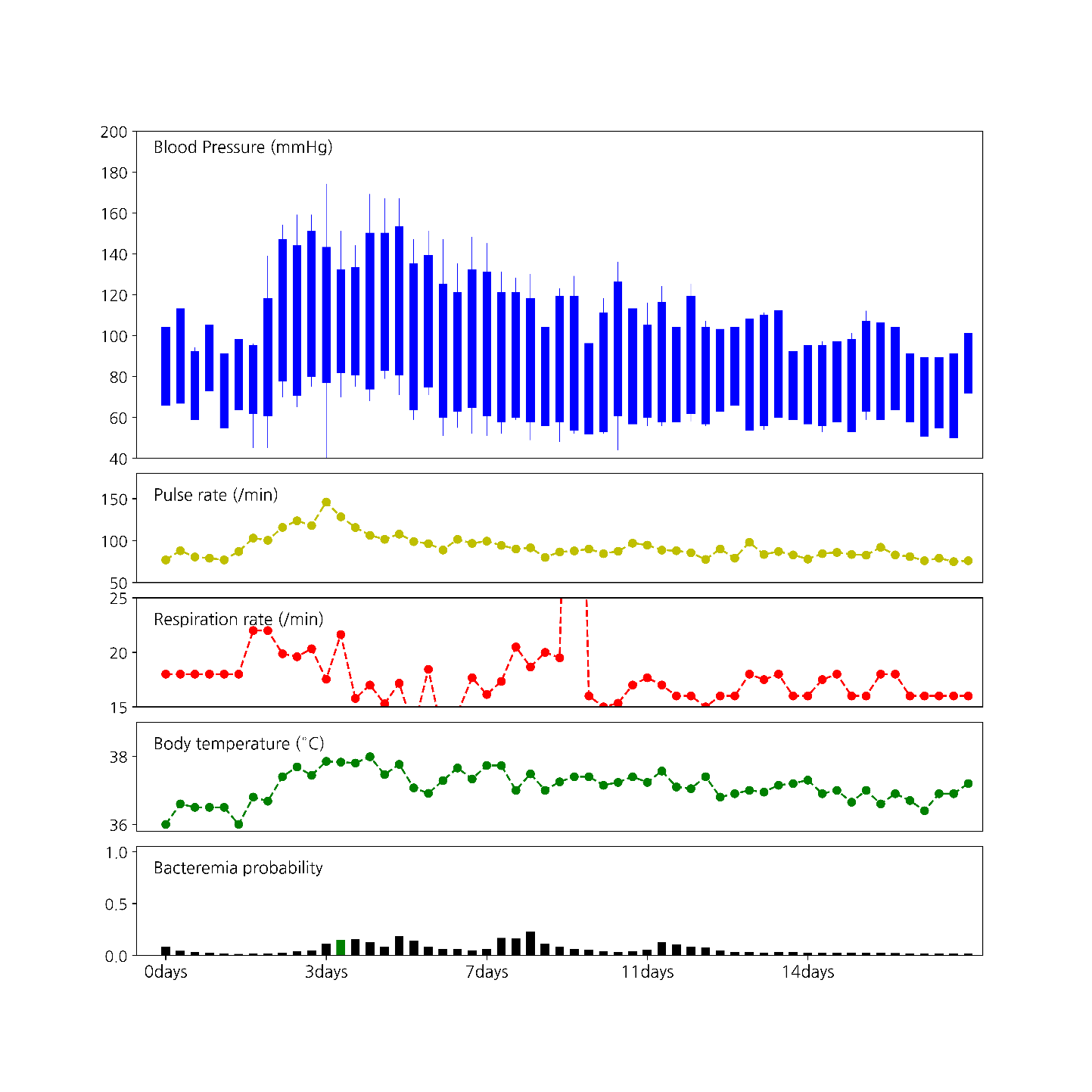

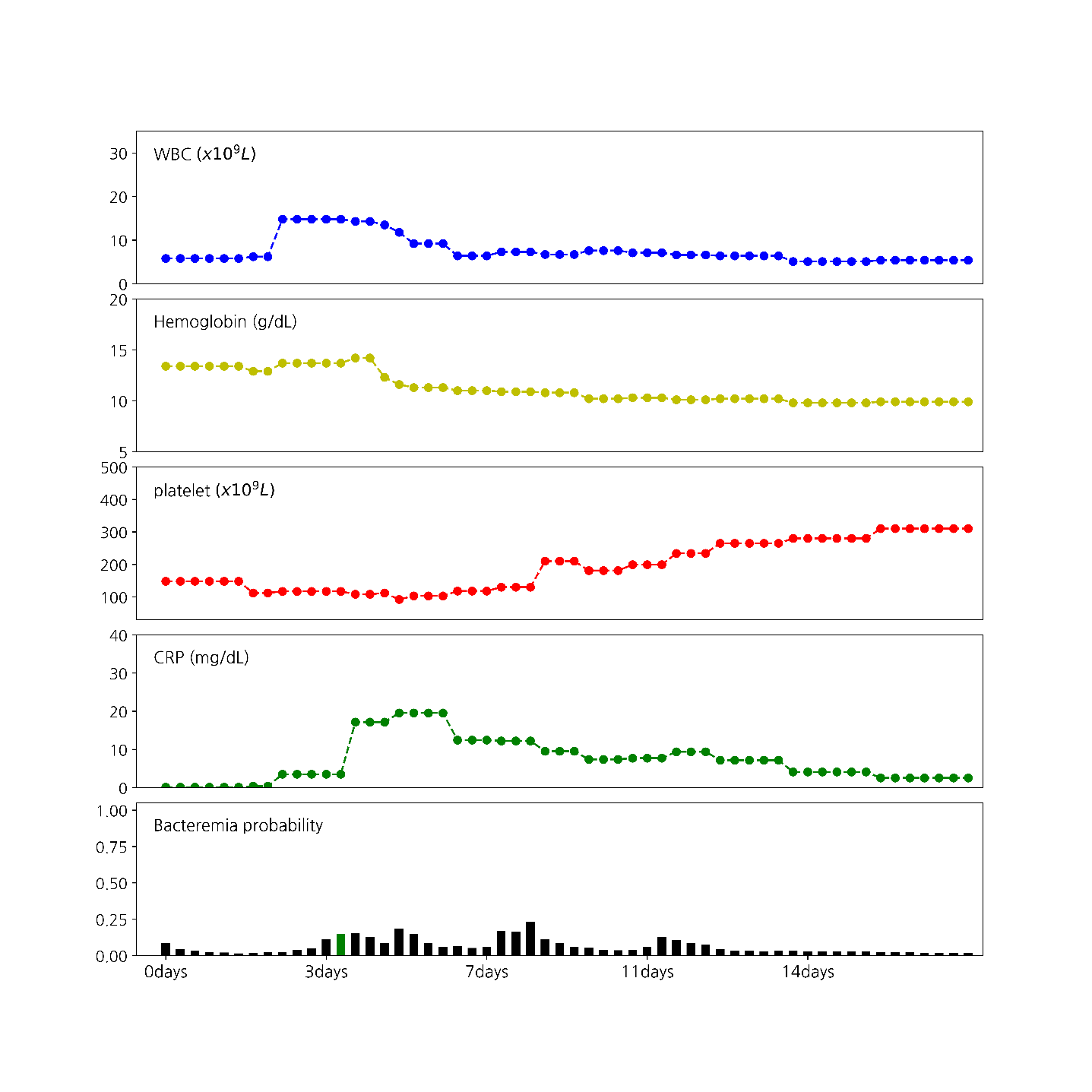


Data from a 76-year-old male admitted for distal common bile duct cancer. The operation was pylorus-preserving pancreaticoduodenectomy performed on hospital day 2. After the operation, the body temperature was elevated along with a high pulse rate. WBC and CRP were also elevated during the period; however, the predicted probability was not increased. On hospital day 3, blood culture was performed (green bar) with no isolation; in this case, blood culture was carried out, but we assumed that the results were not likely to be positive.

4.2 Obscure prediction cases

4.2.1 Obscure prediction case 1


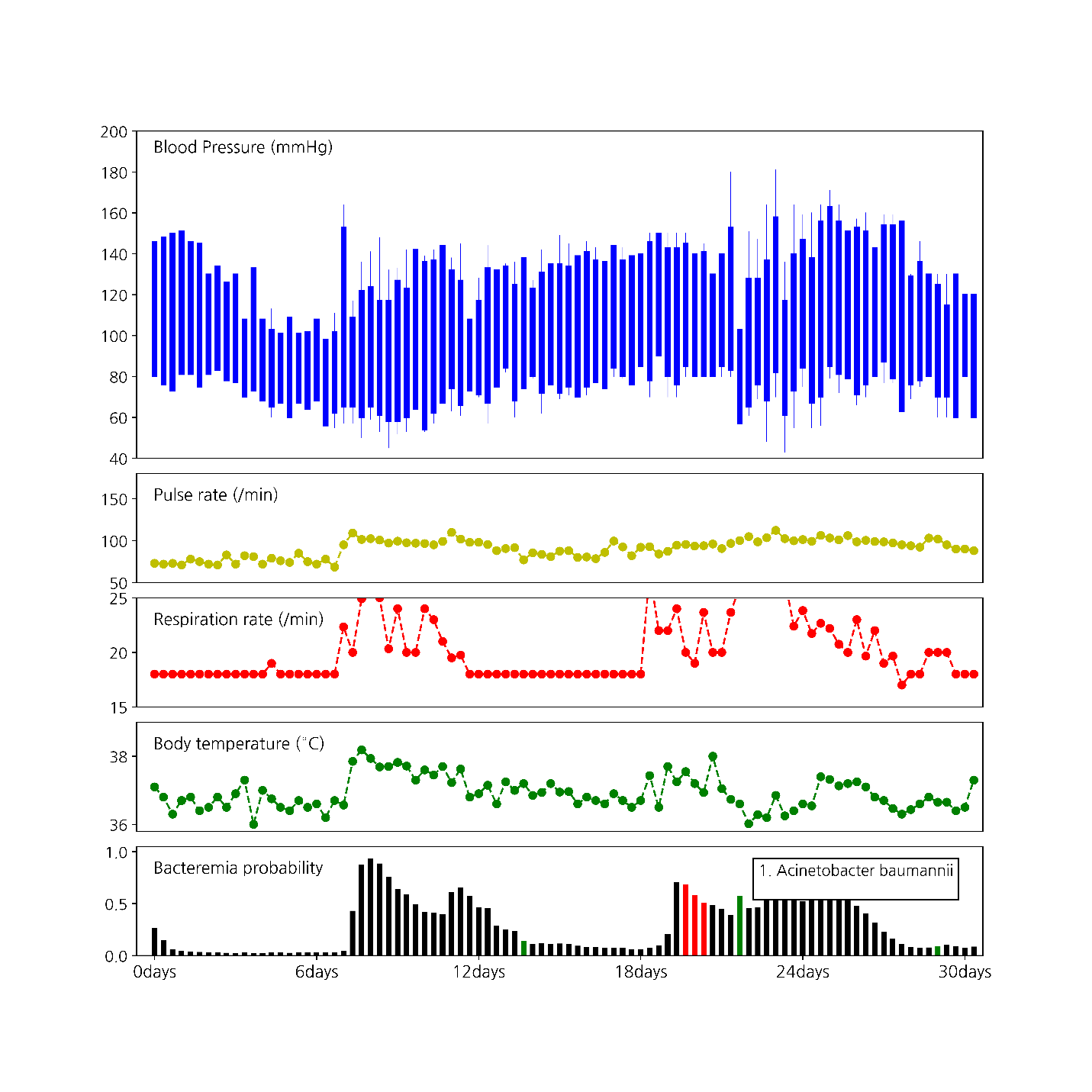

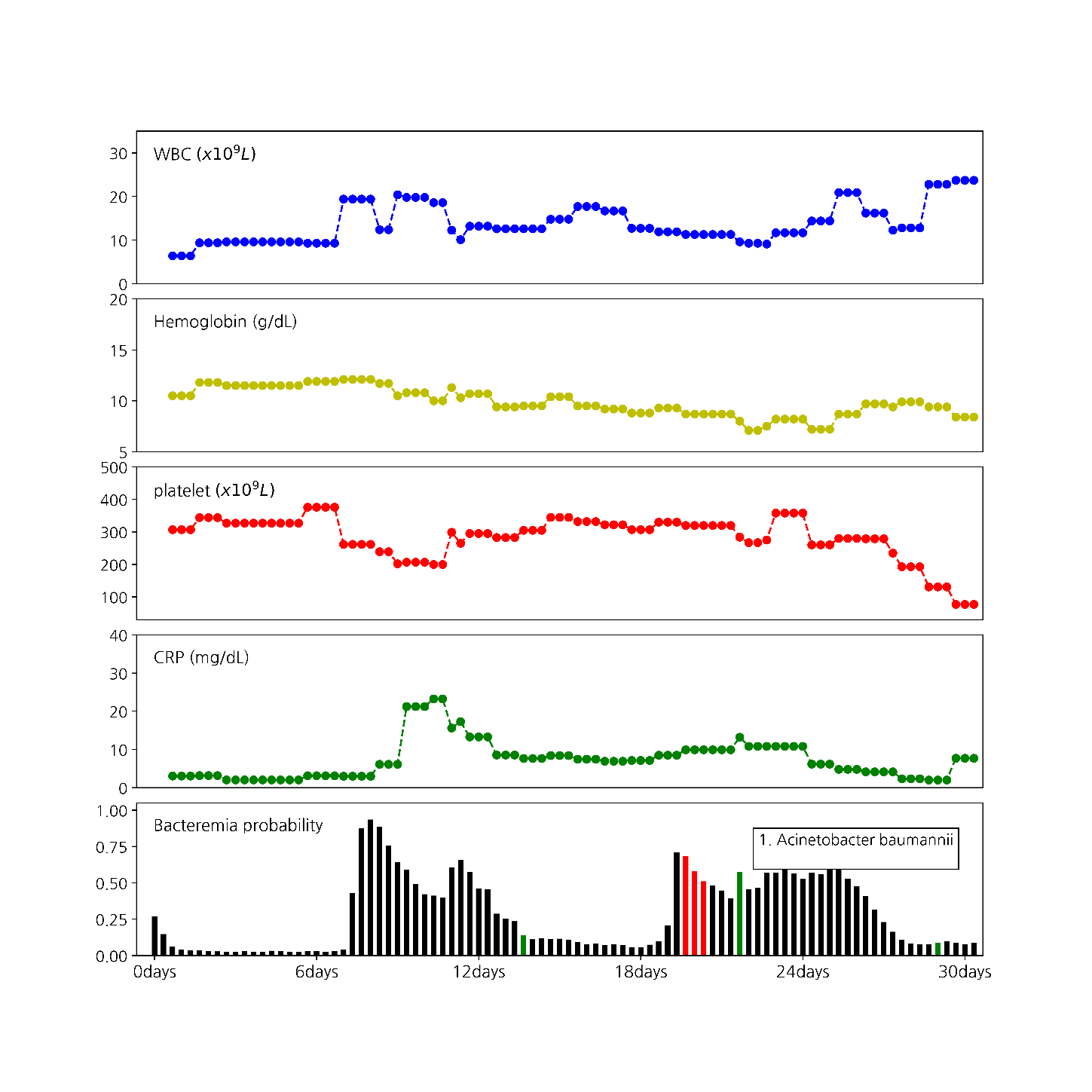


Data from an 81-year-old male patient admitted for cholangiocellular carcinoma. The operation was excision of the bile duct, partial hepatectomy and cholecystectomy performed on hospital day 7. From the hospital day 7 to 12, the predicted probability elevated along with body temperature and respiratory rate, followed by elevated CRP and WBC after 2 to 3 days. If blood culture had been carried out during this period, the result might have been positive considering the high predictive probabilities; however, we cannot be sure because no culture was carried out. On hospital day 13, the blood culture was performed (first green bar), but the prediction probability was around 0.2, and no isolation was found. On hospital day 19, *Acinetobacter baumannii* was isolated and for the next 7 days, the predicted probability was high along with elevated respiratory rate and WBC count.; Green bar: blood culture with no isolation.

4.2.2 Obscure prediction case 2


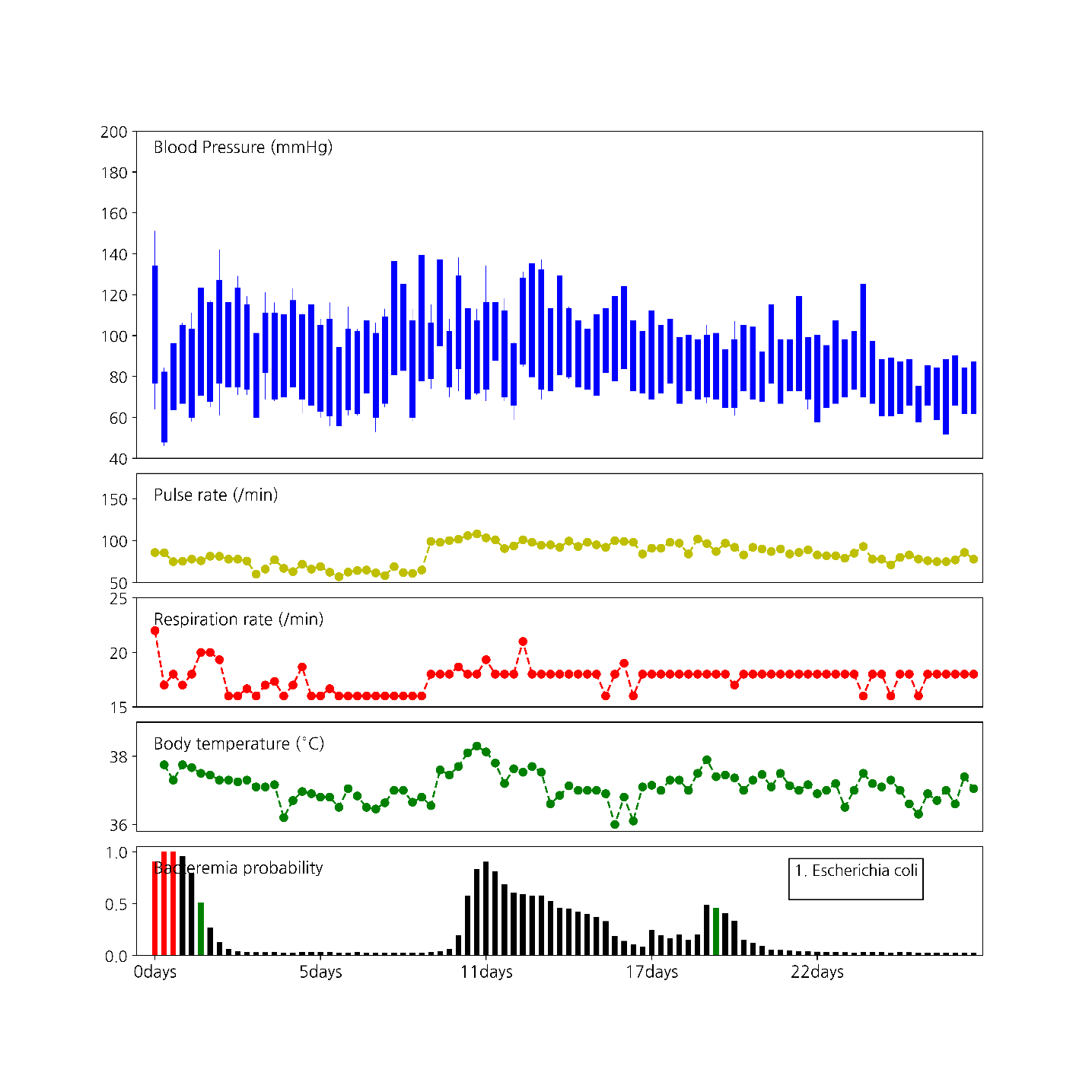

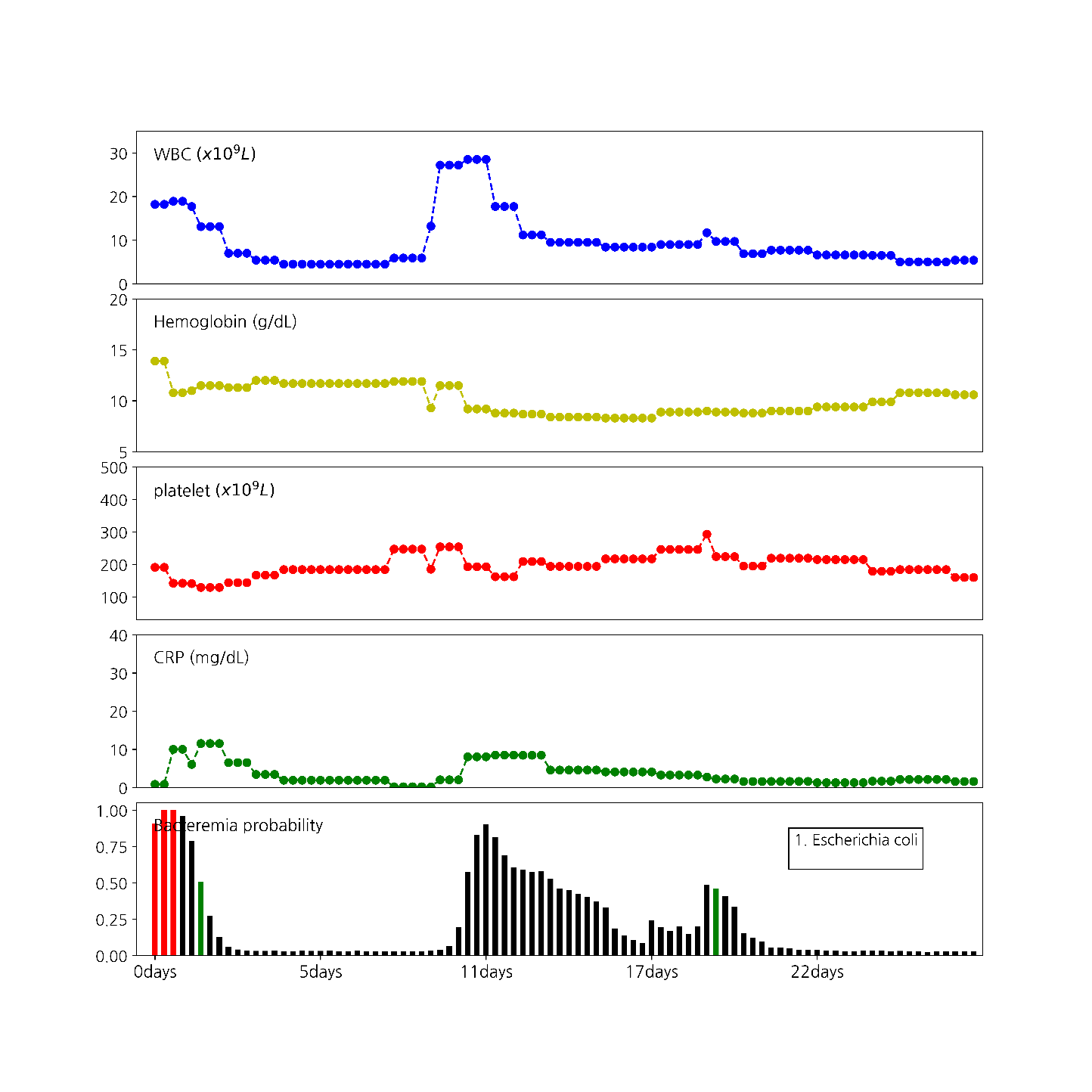


Data from a 66-year-old female patient admitted for intrahepatic duct stone with cholangitis. The operation was liver lobectomy performed on hospital day 10. The patient had been first treated with antibiotics. On the day of operation, the body temperature was elevated along with WBC and pulse rate. Around hospital day 10, the predicted probability was elevated, but blood culture was not performed. In this case, only the first blood culture was positive, isolated with *E. coli*. On hospital day 12, the predictive probability was elevated along with high body temperature and pulse rate; however, the actual presence of bacteremia is unsure because blood culture was not carried out at that time.; Green bar: blood culture with no isolation.

4.2.3 Obscure prediction case 3


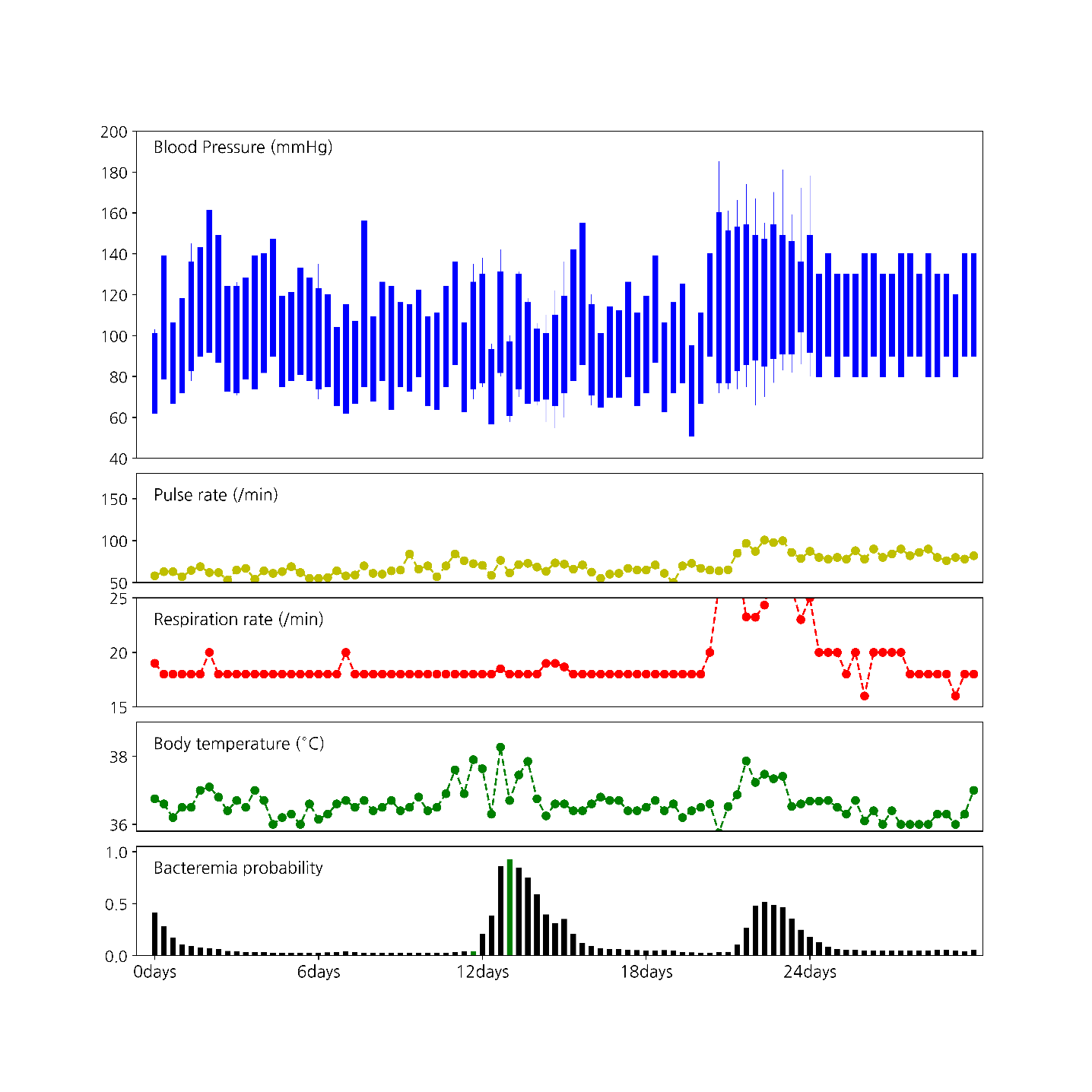

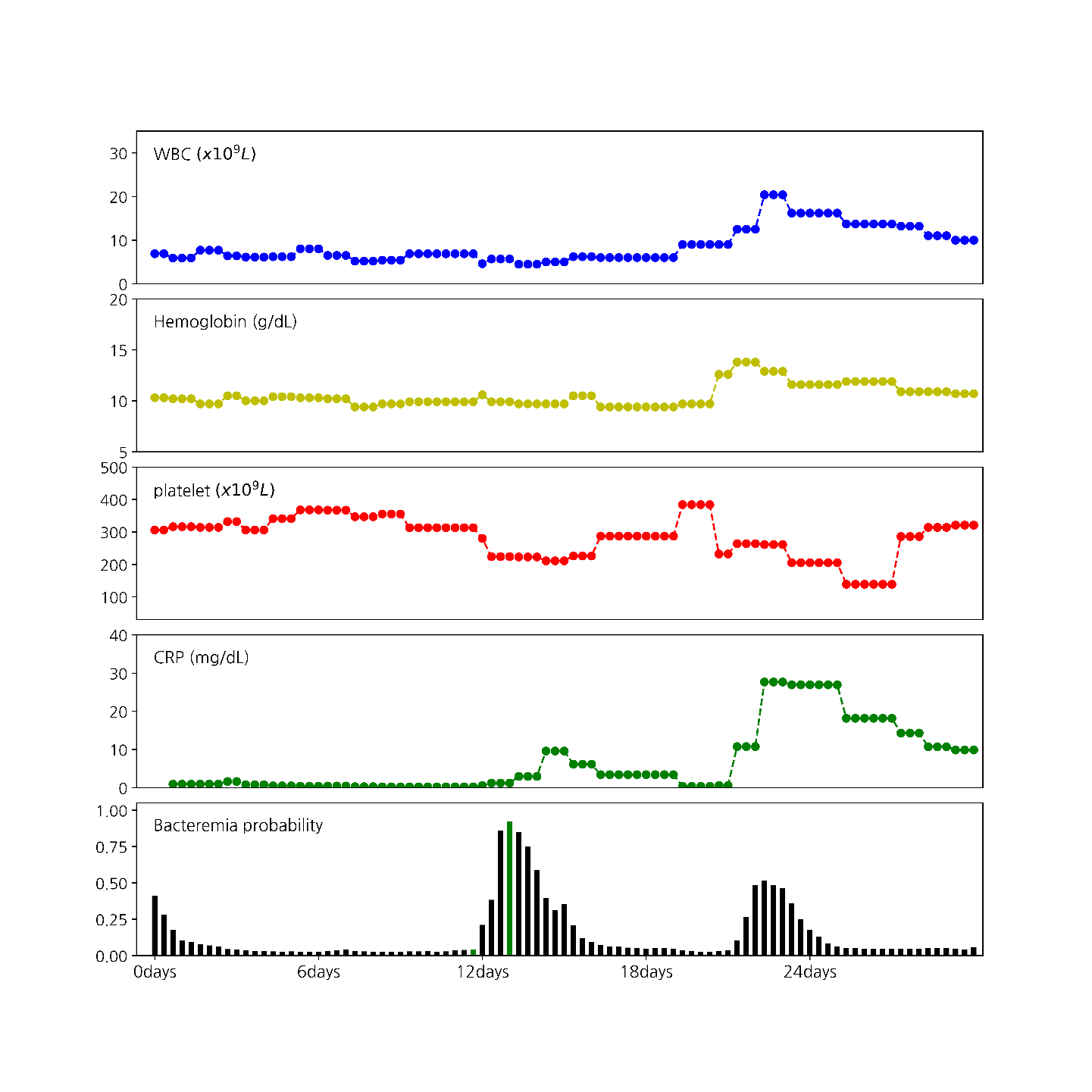


Data from a 76-year-old female patient admitted for common bile duct cancer. Fever was observed on hospital day 13, along with elevated predicted probability. The patient underwent pylorus-preserving pancreatico-duodenectomy at hospital day 21, around which the predicted probability was again elevated. The day with green bar (i.e. blood culture with no isolation) can be associated with infection, or it might be a wrong prediction for bacteremia. Green bar: blood culture with no isolation.

4.3 Bad prediction cases

4.3.1 Bad prediction case 1.


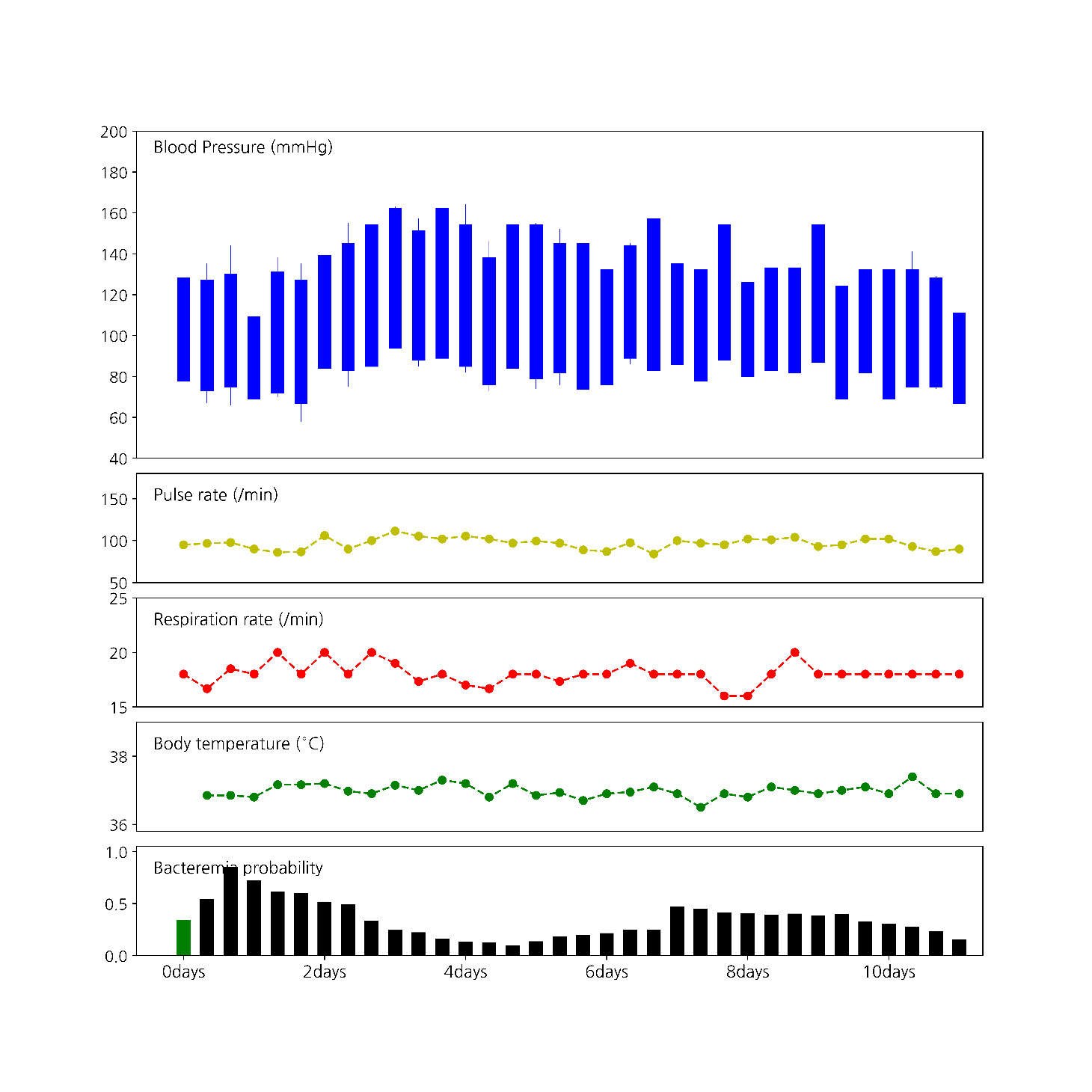

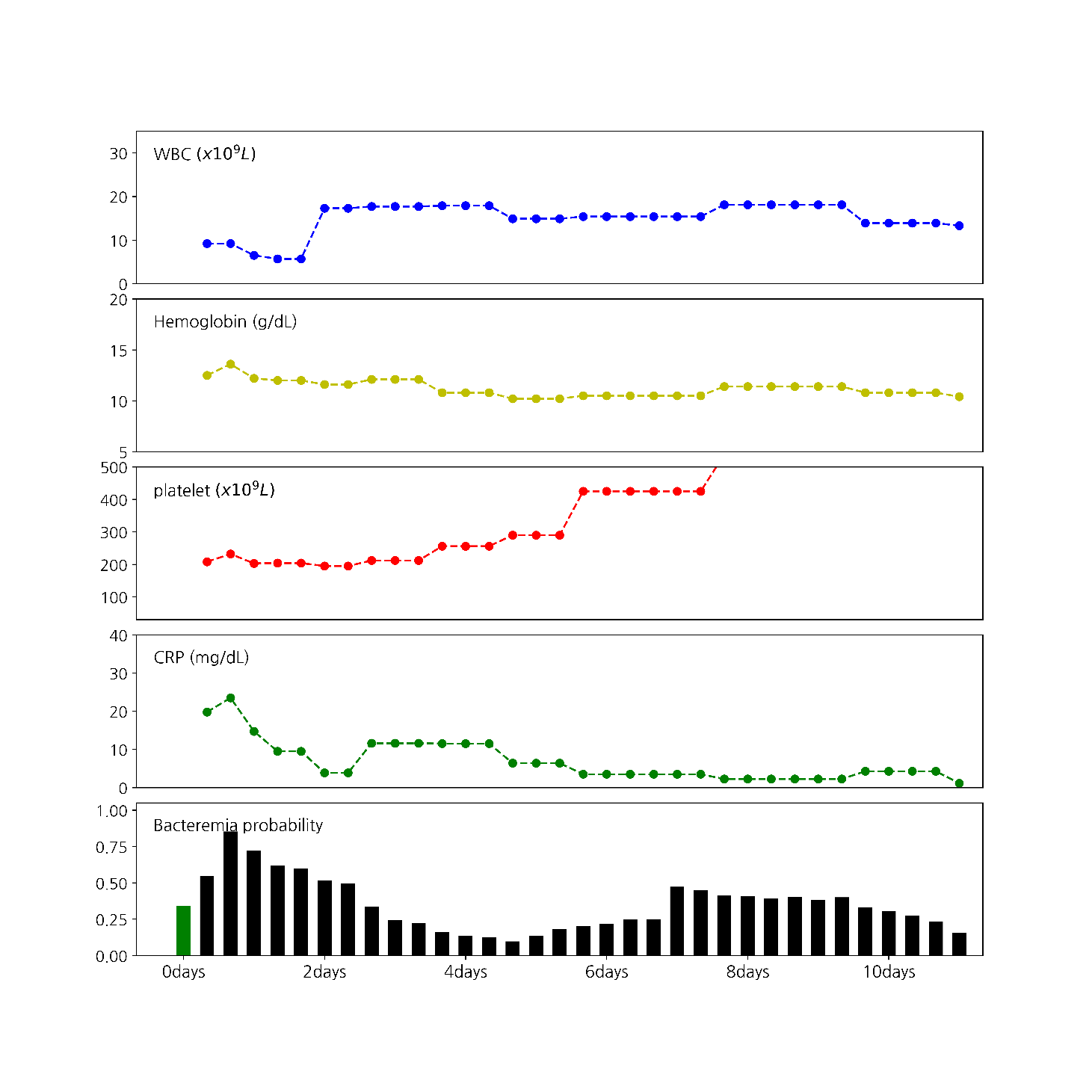


Data from a 71-year-old male patient admitted for pancreas cancer. Blood culture was performed at admission and the patient underwent a Whipple's operation on hospital day 2. Although CRP was gradually stabilized, and fever was not identified, the predicted probability was elevated during the whole period of admission.; Green bar: blood culture with no isolation.

Supplement e-Figure 5. Example of the transformation of the skewed data.


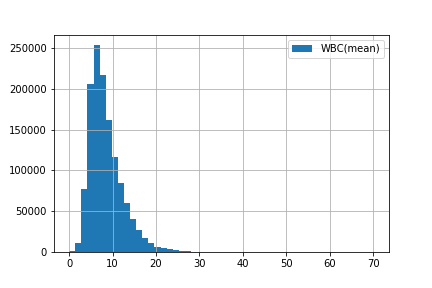

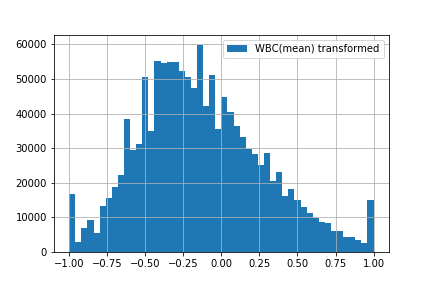

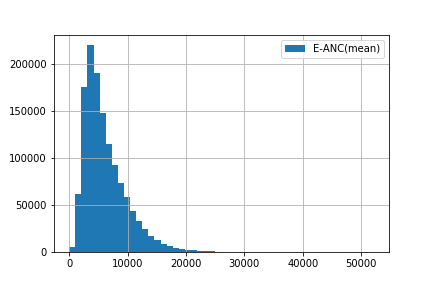

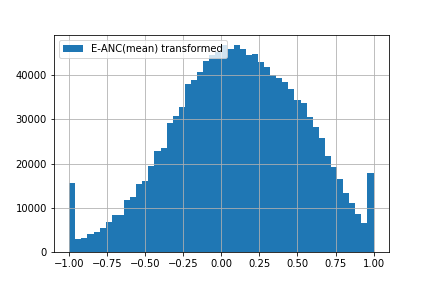

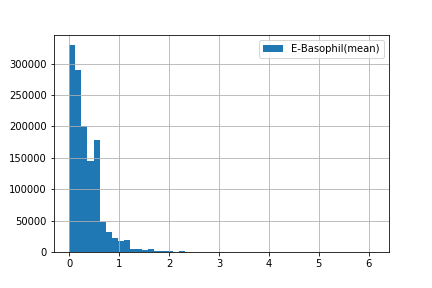

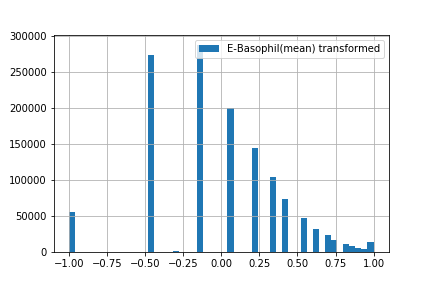

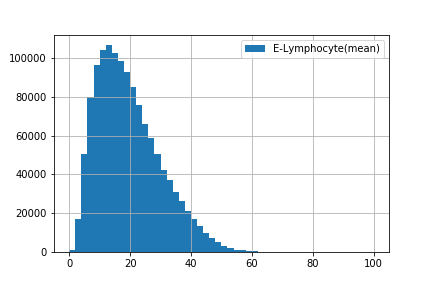

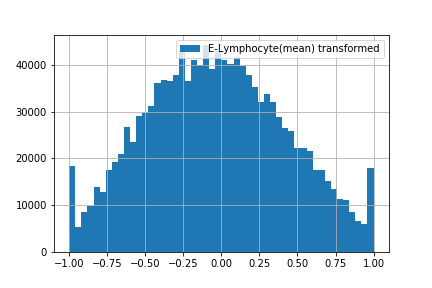

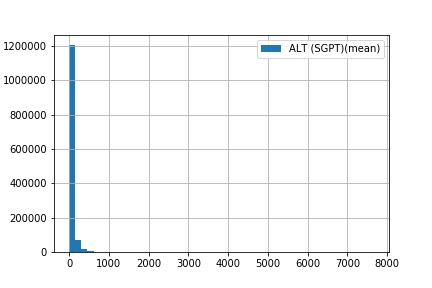

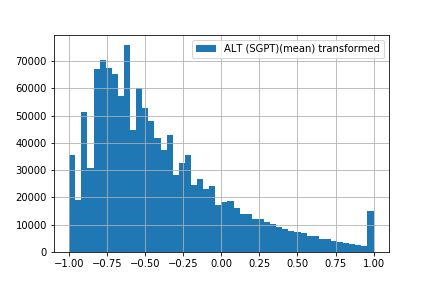

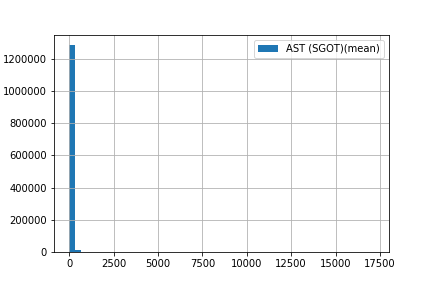

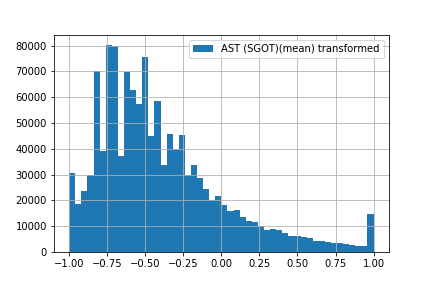

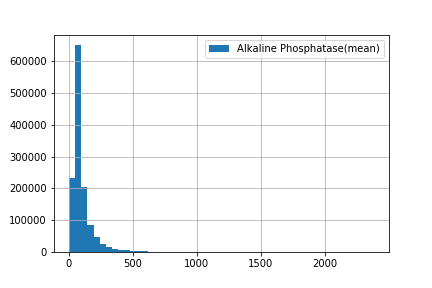

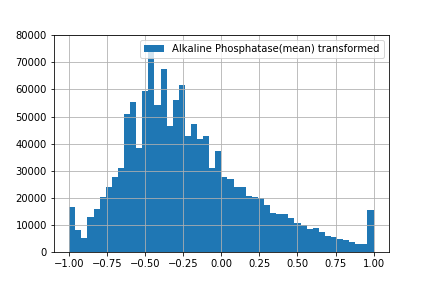

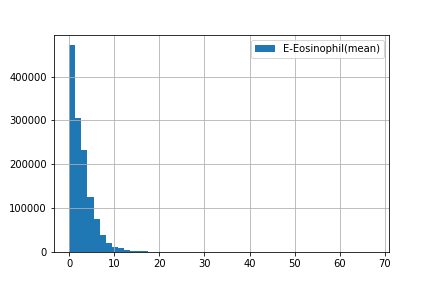

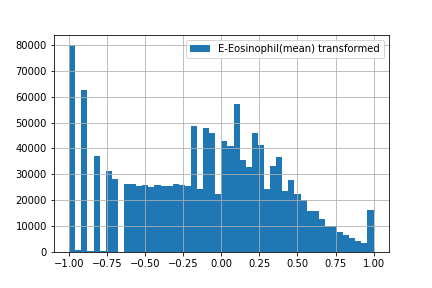

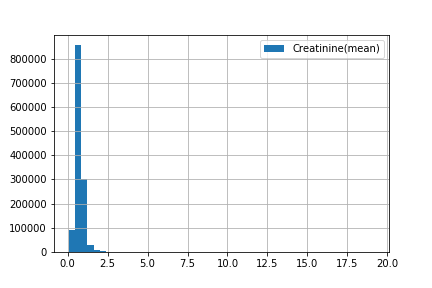

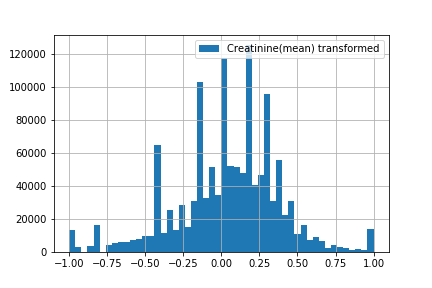


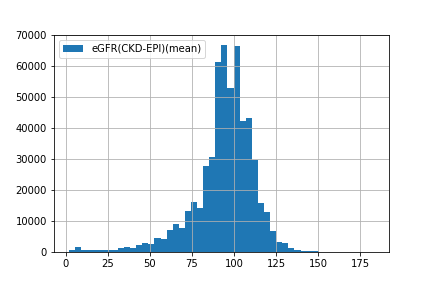

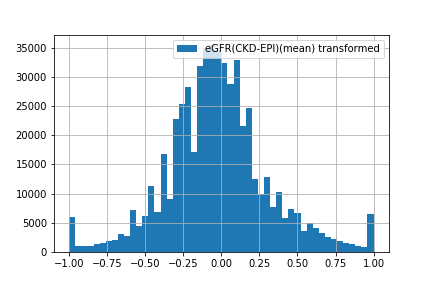


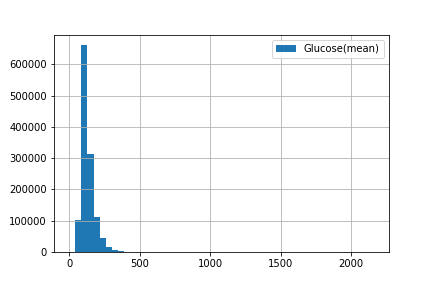

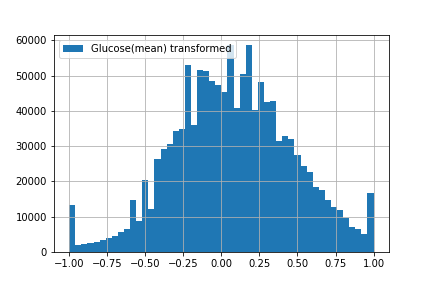

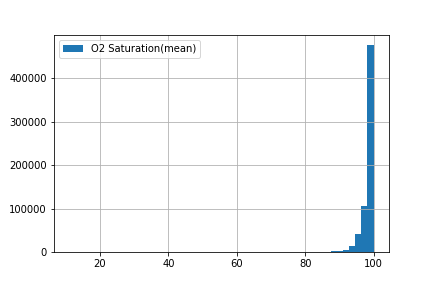

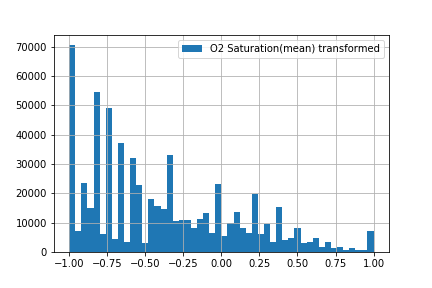
 The left plots are raw distributions of data. The right plots are the transformed distributions from Box-Cox transformation. The parameters used in the Box-Cox transformer were based on the skewness of data.

**Section 5. Reference**

1. Sphinx. Regular expression operation [Internet]. 2020 [cited 2020 Jan 19]. Available from: https://docs.python.org/3/library/re.html

2. Tomašev N, Glorot X, Rae JW, Zielinski M, Askham H, Saraiva A, Mottram A, Meyer C, Ravuri S, Protsyuk I, Connell A, Hughes CO, Karthikesalingam A, Cornebise J, Montgomery H, Rees G, Laing C, Baker CR, Peterson K, Reeves R, Hassabis D, King D, Suleyman M, Back T, Nielson C, Ledsam JR, Mohamed S. A clinically applicable approach to continuous prediction of future acute kidney injury. Nature [Internet] Springer US; 2019;572(7767):116–119. [doi: 10.1038/s41586-019-1390-1]

3. Mao Q, Jay M, Hoffman JL, Calvert J, Barton C, Shimabukuro D, Shieh L, Chettipally U, Fletcher G, Kerem Y, Zhou Y, Das R. Multicentre validation of a sepsis prediction algorithm using only vital sign data in the emergency department, general ward and ICU. BMJ Open England; 2018 Jan;8(1):e017833. PMID:29374661

4. Sakia RM. The Box-Cox transformation technique: a review. 1992;169–178.

5. Fu R, Zhang Z, Li L. Using LSTM and GRU neural network methods for traffic flow prediction. 2016 31st Youth Acad Annu Conf Chinese Assoc Autom 2016. p. 324–328. [doi: 10.1109/YAC.2016.7804912]

6. Hochreiter S. Long Short-term Memory. 2016;(December 1997). [doi: 10.1162/neco.1997.9.8.1735]

7. Chung J, Gulcehre C, Cho K, Bengio Y. Empirical Evaluation of Gated Recurrent Neural Networks on Sequence Modeling. 2014;1–9.

8. Glorot X, Bengio Y. Understanding the difficulty of training deep feedforward neural networks. J Mach Learn Res 2010;9:249–256.

9. Kingma DP, Ba JL. Adam: A Method for Stochastic Optimization. 2015;1–15.

10. Singer M, Deutschman CS, Seymour C, Shankar-Hari M, Annane D, Bauer M, Bellomo R, Bernard GR, Chiche JD, Coopersmith CM, Hotchkiss RS, Levy MM, Marshall JC, Martin GS, Opal SM, Rubenfeld GD, Poll T Der, Vincent JL, Angus DC. The third international consensus definitions for sepsis and septic shock (sepsis-3). JAMA - J Am Med Assoc 2016;315(8):801–810. PMID:26903338

11. Shankar-Hari M, Harrison DA, Rubenfeld GD, Rowan K. Epidemiology of sepsis and septic shock in critical care units: Comparison between sepsis-2 and sepsis-3 populations using a national critical care database. Br J Anaesth [Internet] The Author(s); 2017;119(4):626–636. [doi: 10.1093/bja/aex234]

12. Tajima Y, Tsuruta M, Yahagi M, Hasegawa H, Okabayashi K, Shigeta K, Ishida T, Kitagawa Y. Is preoperative spirometry a predictive marker for postoperative complications after colorectal cancer surgery? Jpn J Clin Oncol 2017;47(9):815–819. [doi: 10.1093/jjco/hyx082]

13. Kim TH, Lee JS, Lee SW, Oh YM. Pulmonary complications after abdominal surgery in patients with mild-to-moderate chronic obstructive pulmonary disease. Int J COPD 2016;11(1):2785–2796. [doi: 10.2147/COPD.S119372]
